# Supplementary material for: Interspecific variation in the limb long bones among modern rhinoceroses—extent and drivers
Source: PeerJ. 2019 Sep 26;7:e7647. doi: 10.7717/peerj.7647 (PMC6766374; doi:10.7717/peerj.7647)
Supplement: Supplemental Information 1 [file peerj-07-7647-s001.pdf]

Data S1: Designation and location of anatomical landmarks placed on each bone.

Bone	Anatomical LM	Curve sliding semi-LM	Surface sliding semi-LM	Total
**Humerus**	35	639	1437	2111
**Radius**	23	393	920	1336
**Ulna**	21	343	822	1142
**Femur**	27	612	1031	1670
**Tibia**	24	384	854	1262
**Fibula**	12	269	454	735

Table S1A: Total number of anatomical landmarks (LM), curve sliding and surface sliding semi-landmarks for each bone.

LM	Designation
**1**	Most distal point of the lateral border of the bicipital groove
**2**	Most proximal point of the lateral border of the bicipital groove
**3**	Most proximal point of the intermediate tubercle
**4**	Most proximal point of the medial border of the bicipital groove
**5**	Most distal point of the medial border of the bicipital groove
**6**	Most distal point of the intermediate tubercle
**7**	Most medial point of the top of the lesser tubercle
**8**	Most cranial point of the lesser tubercle convexity
**9**	Most medio-caudal point of the lesser tubercle convexity
**10**	Most medial point of the humeral head surface
**11**	Most caudo-distal point of the humeral head surface
**12**	Contact point between the tricipital line and the caudal border of the articular head surface
**13**	Most lateral point of the humeral head surface
**14**	Most caudal point of the greater tubercle convexity
**15**	Most proximal point of the greater tubercle convexity
**16**	Most cranial point of the greater tubercle convexity crest
**17**	Most proximal point of the m. infraspinatus lateral insertion
**18**	Most distal point of the m. infraspinatus lateral insertion
**19**	Most proximal point of the deltoid tuberosity
**20**	Most distal point of the deltoid tuberosity
**21**	Most proximal point of the epicondylar crest tuberosity
**22**	Most distal point of the epicondylar crest tuberosity
**23**	Most lateral point of the lateral epicondyle
**24**	Most distal point of the lateral epicondyle
**25**	Most proximo-lateral point of the capitulum
**26**	Most cranio-proximal point of contact between the trochlea and the capitulum
**27**	Most cranial point of the trochlea groove
**28**	Most cranio-medial point of the dorsal side of the trochlea
**29**	Most distal contact point between the trochlea border and the medial development of the trochlea lip
**30**	Most cranio-medial point of the ventral side of the trochlea
**31**	Most cranio-lateral point of the ventral side of the trochlea
**32**	Most caudo-distal point of contact between the capitulum and the trochlea
**33**	Most medial point of the medial epicondyle
**34**	Most caudal point of the medial epicondyle
**35**	Most lateral point of the medial epicondyle

Table S1B: Designation of anatomical landmarks on the humerus.

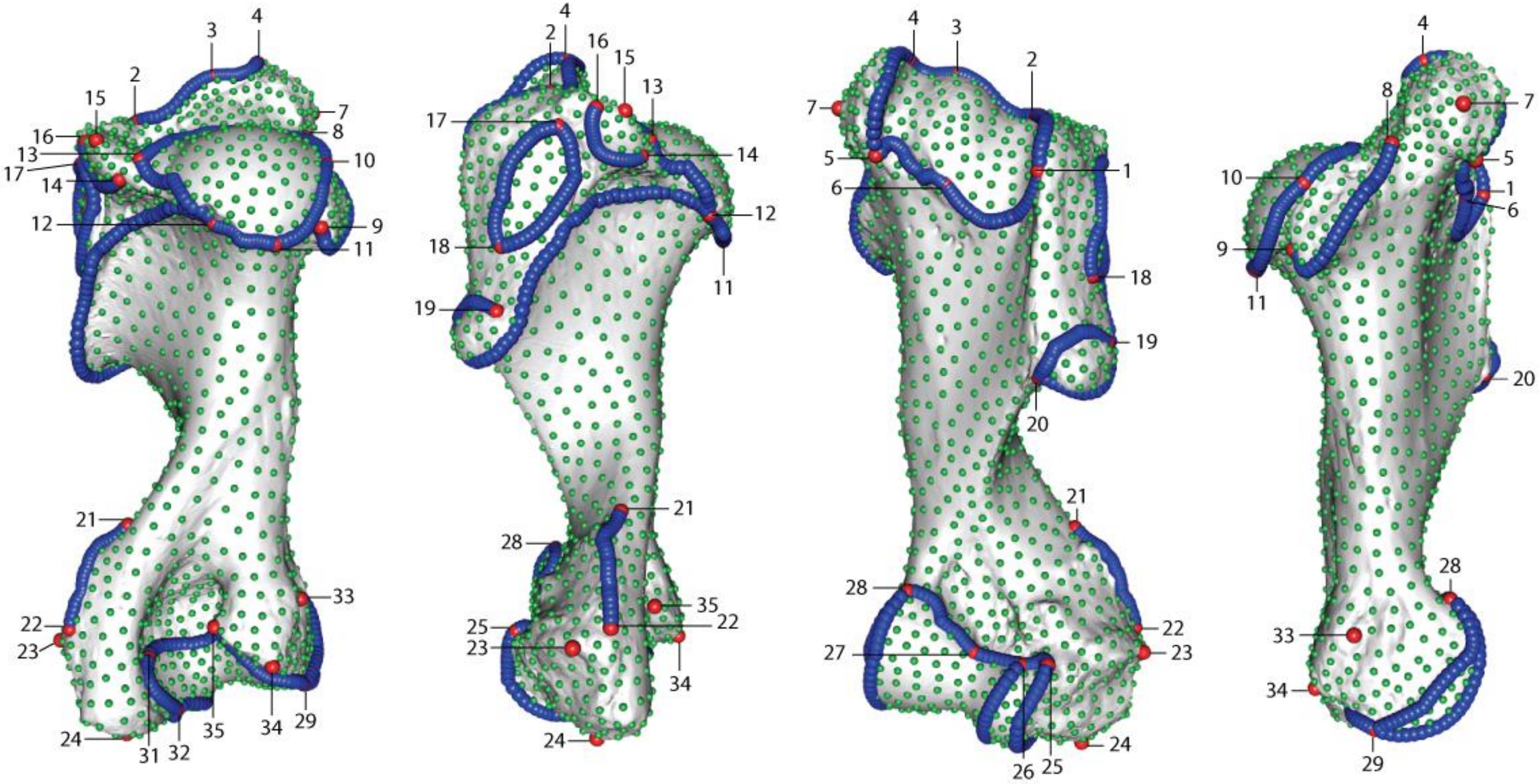

Figure S1C: Location of anatomical landmarks (red spheres), curve sliding (blue spheres) and surface sliding (green spheres) semi-landmarks placed on the humerus. From left to right: caudal, lateral, cranial and medial views. Numbers refer to anatomical landmarks designation detailed in Table S1B. Landmark n°30 situated in the olecranon fossa cannot be seen.

LM	Designation
**1**	Most caudo-lateral point of the lateral glenoid cavity
**2**	Most cranio-lateral point of the lateral glenoid cavity
**3**	Tip of the coronoid process
**4**	Most cranial point of the medial glenoid cavity
**5**	Most caudo-medial point of the medial glenoid cavity
**6**	Tip of the palmar process of the glenoid cavity ridge
**7**	Most cranial point of the lateral insertion relief
**8**	Most lateral point of the lateral insertion relief
**9**	Most caudo-distal point of the proximo-lateral articular facet for the ulna
**10**	Most caudo-distal point of the proximo-medial articular facet for the ulna
**11**	Most proximal point of the interosseous crest (= most distal point of the interosseous space)
**12**	Most distal point of the interosseous crest (crossing the distal epiphysis line)
**13**	Most cranio-lateral point of the disto-lateral articulation surface for ulna
**14**	Most proximo-lateral point of the disto-lateral articulation surface for ulna
**15**	Most caudo-lateral point of the disto-lateral articulation surface for ulna
**16**	Most medial point of the transversal crest
**17**	Tip of the radial styloid process
**18**	Maximum of curvature of the cranial ridge of the articular facet for the scaphoid
**19**	Most cranio-lateral point of the articular facet for the scaphoid
**20**	Most lateral point of the articular facet for the semilunar
**21**	Most caudo-lateral point of the articular facet for the semilunar
**22**	Most caudo-lateral point of the articular facet for the scaphoid
**23**	Most cranio-proximal point of the medial facet of distal radius

Table S1D: Designation of anatomical landmarks on the radius.

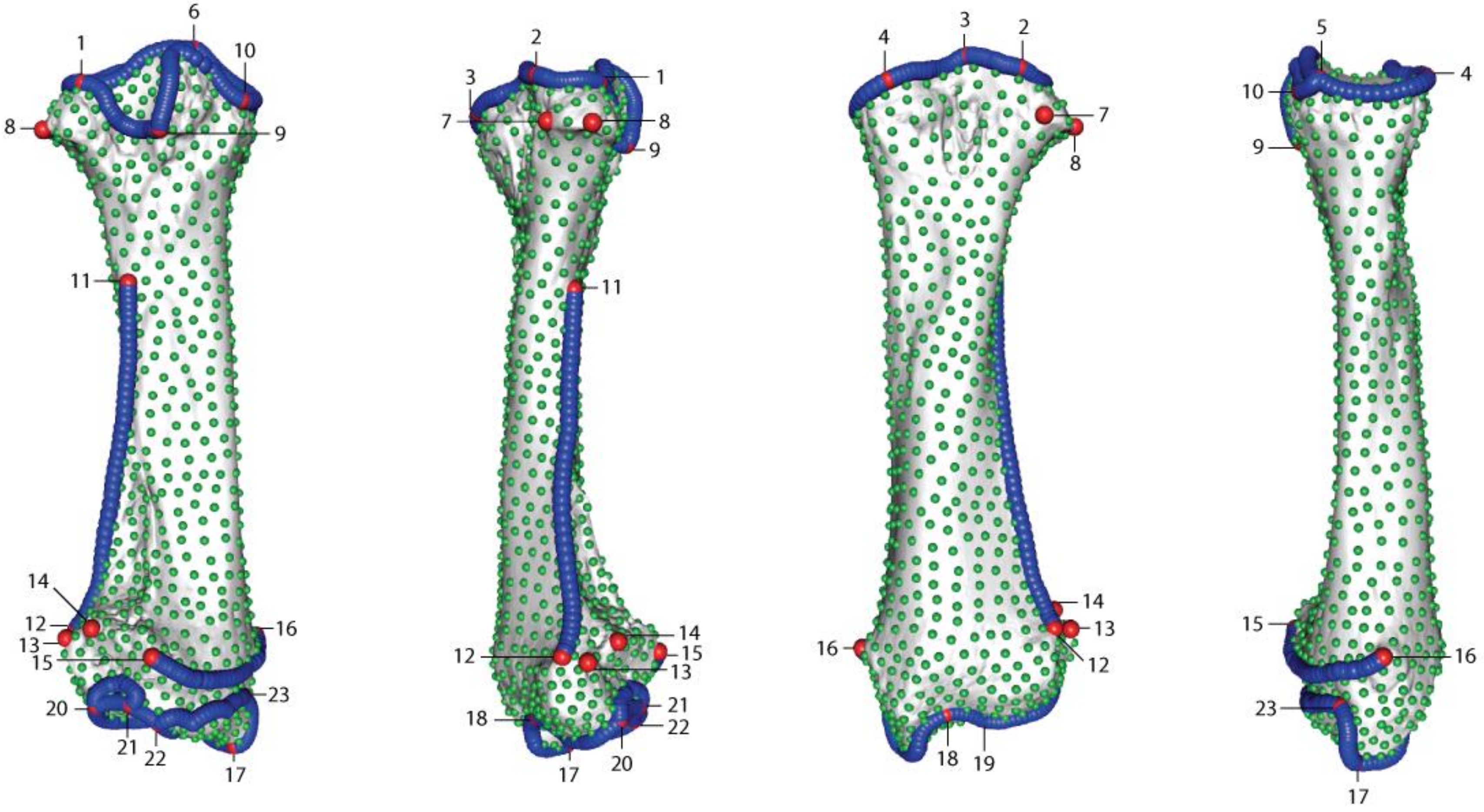

Figure S1E: Location of anatomical landmarks (red spheres), curve sliding (blue spheres) and surface sliding (green spheres) semi-landmarks placed on the radius. From left to right: caudal, lateral, cranial and medial views. Numbers refer to anatomical landmarks designation detailed in Table S1D.

LM	Designation
**1**	Most proximo-cranial point of the olecranon tuberosity cranial border
**2**	Most lateral point of the olecranon tuberosity
**3**	Most caudo-distal point of the olecranon tuberosity
**4**	Most medial point of the olecranon tuberosity
**5**	Most proximal point of the olecranon tuberosity
**6**	Cranial tip of the anconeal process
**7**	Most distal point of the lateral part of the trochlear notch articular surface
**8**	Maximum concavity point of the distal border of the trochlear notch articular surface
**9**	Most distal point of the medial part of the trochlear notch articular surface
**10**	Most distal point of the proximo-medial articular facet for the radius
**11**	Most distal point of the proximo-lateral articular facet for the radius
**12**	Most distal point of the proximal synostosis surface for the radius (= most proximal point of the interosseous space)
**13**	Most medio-caudal point of the distal radio-ulnar synostosis surface
**14**	Most disto-medial point of the articular surface with the semilunar bone
**15**	Most cranio-lateral point of the articular surface with the semilunar bone
**16**	Most disto-lateral point of the articular surface with the semilunar bone
**17**	Most cranio-lateral point of the distal radio-ulnar synostosis surface
**18**	Most lateral point of the distal epiphysis
**19**	Caudo-distal tip of ulnar styloid process
**20**	Most proximal contact point between the articular surfaces for the pisiform and the triquetrum
**21**	Most distal contact point between the caudal border of the ulna and the articular surface with the pisiform

Table S1F: Designation of anatomical landmarks on the ulna.

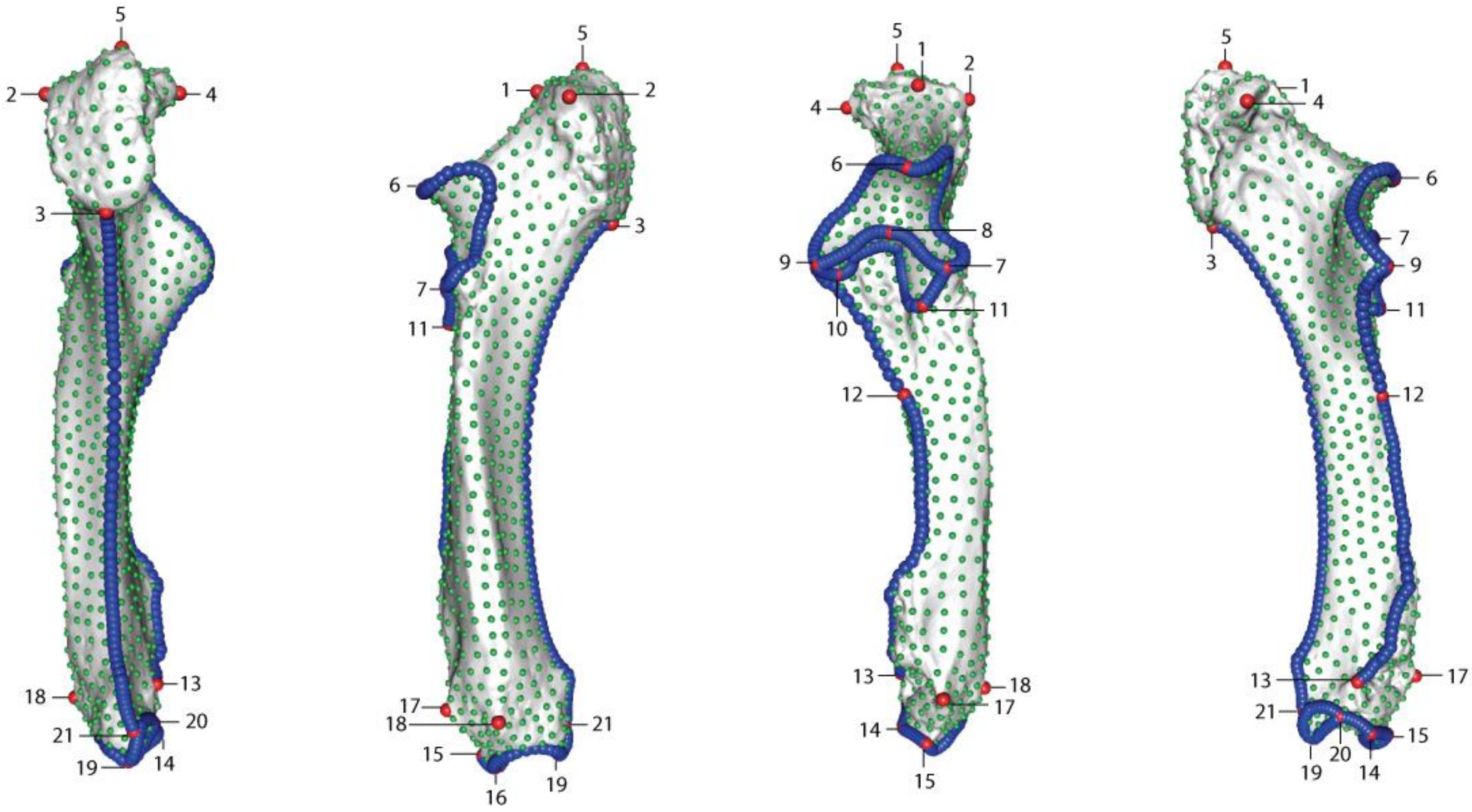

Figure S1G: Location of anatomical landmarks (red spheres), curve sliding (blue spheres) and surface sliding (green spheres) semi-landmarks placed on the ulna. From left to right: caudal, lateral, cranial and medial views. Numbers refer to anatomical landmarks designation detailed in Table S1F.

LM	Designation
**1**	Most proximo-cranial point of the greater trochanter
**2**	Most proximo-caudal point of the greater trochanter
**3**	Most medial point of the greater trochanter convexity
**4**	Most distal point of the intertrochanteric crest
**5**	Most disto-caudal point of the greater trochanter
**6**	Most cranio-lateral point of the convexity of the greater trochanter
**7**	Most proximal contact point between the intertrochanteric line and the medial line of the cranial face
**8**	Most lateral point of the border of the head
**9**	Most proximal point of the lesser trochanter
**10**	Most distal point of the lesser trochanter
**11**	Most proximal point of the gluteal tuberosity on the third trochanter
**12**	Most distal point of the gluteal tuberosity on the third trochanter
**13**	Most medial point of the medial epicondyle
**14**	Contact point between the intercondylar line and the medial condyle
**15**	Contact point between the intercondylar line and the lateral condyle
**16**	Most lateral point of the lateral epicondyle
**17**	Most proximal point of the lateral lip of the trochlea
**18**	Most proximal point of the trochlear groove
**19**	Most proximal point of the medial lip of the trochlea
**20**	Most distal point of the medial lip of the trochlea
**21**	Distal maximum of curvature of the trochlear groove
**22**	Most distal point of the lateral lip of the trochlea
**23**	Most medial point of the fossa extensoria
**24**	Most lateral point of the fossa extensoria
**25**	Most cranial point of the fossa extensoria
**26**	Most proximo-medial point of the lateral condyle articular surface
**27**	Most proximo-lateral point of the medial condyle articular surface

Table S1H: Designation of anatomical landmarks on the femur.

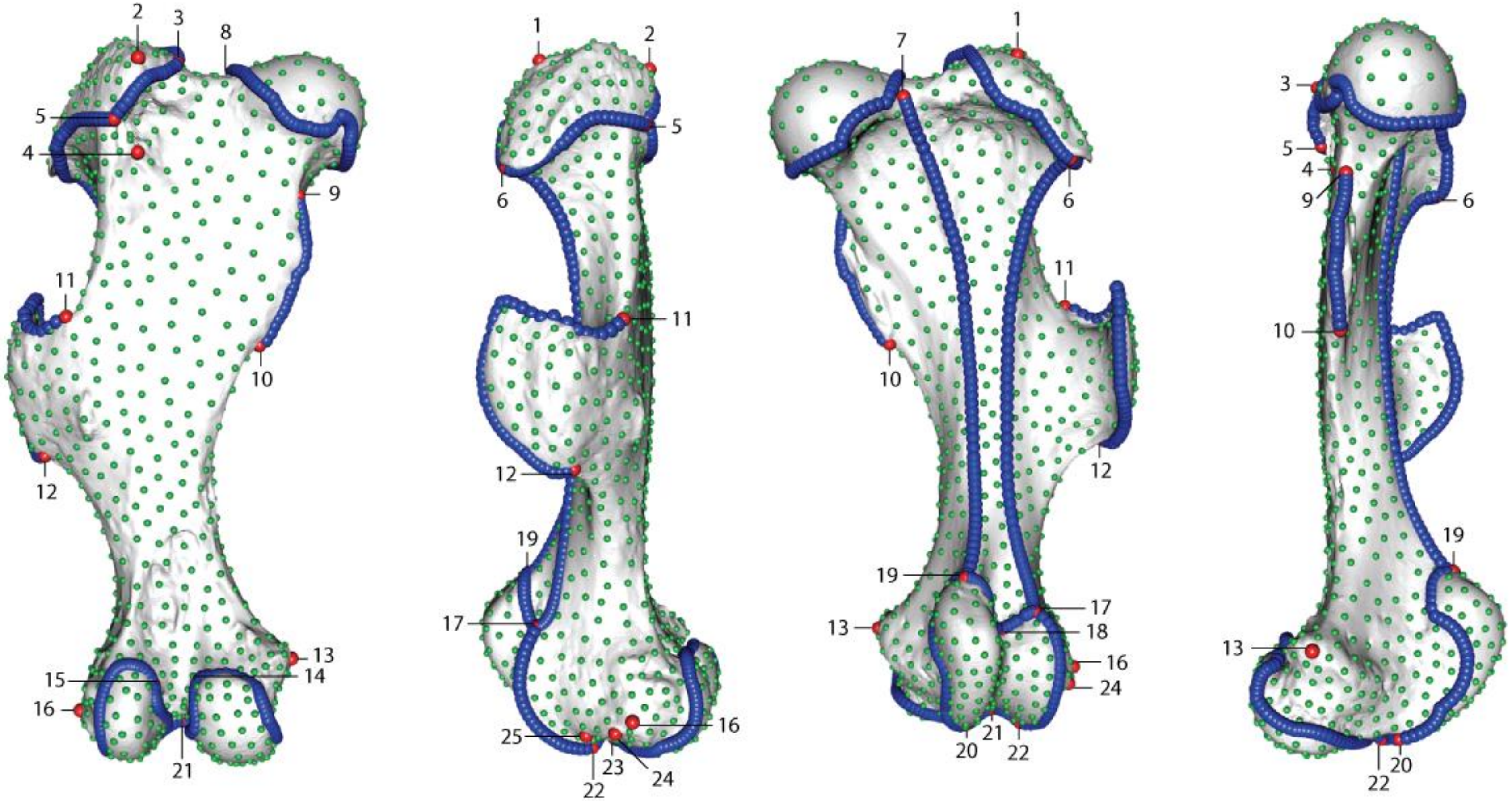

Figure S1I: Location of anatomical landmarks (red spheres), curve sliding (blue spheres) and surface sliding (green spheres) semi-landmarks placed on the femur. From left to right: caudal, lateral, cranial and medial views. Numbers refer to anatomical landmarks designation detailed in Table S1H. Landmarks n°26 and 27 situated in the intercondylar space cannot be seen.

LM	Designation
**1**	Most proximal point of the lateral tubercle of the intercondylar eminence
**2**	Most proximo-cranial point of the lateral tubercle of the intercondylar eminence
**3**	Most cranial point of the articular surface of the lateral condyle
**4**	Most caudal point of the articular surface of the lateral condyle
**5**	Most caudal point of the lateral tubercle of the intercondylar eminence
**6**	Most caudo-proximal point of the medial tubercle of the intercondylar eminence
**7**	Most proximal point of the medial tubercle of the intercondylar eminence
**8**	Most cranial point of the articular surface of the medial condyle
**9**	Most caudal point of the articular surface of the medial condyle
**10**	Most proximal point of the proximal tibio-fibular synostosis surface
**11**	Most distal point of the proximal tibio-fibular synostosis surface
**12**	Most proximal point of the lateral part of the tibial tuberosity
**13**	Most distal point of the lateral part of the tibial tuberosity
**14**	Most distal point of the tibial tuberosity groove
**15**	Most proximal point of the medial part of the tibial tuberosity
**16**	Most caudal point of the medial condyle
**17**	Most proximal point of the distal tibio-fibular synostosis surface
**18**	Most caudo-lateral point of the distal articular surface
**19**	Most cranio-lateral point of the distal articular surface
**20**	Most cranio-distal point of the intermediate ridge of the distal articular surface
**21**	Most distal point of the contact between the medial malleolus and the distal articular surface
**22**	Most distal point of the medial part of the distal articular surface
**23**	Most caudo-distal point of the intermediate ridge of the distal articular surface
**24**	Most medial point of the medial malleolus

Table S1J: Designation of anatomical landmarks on the tibia.

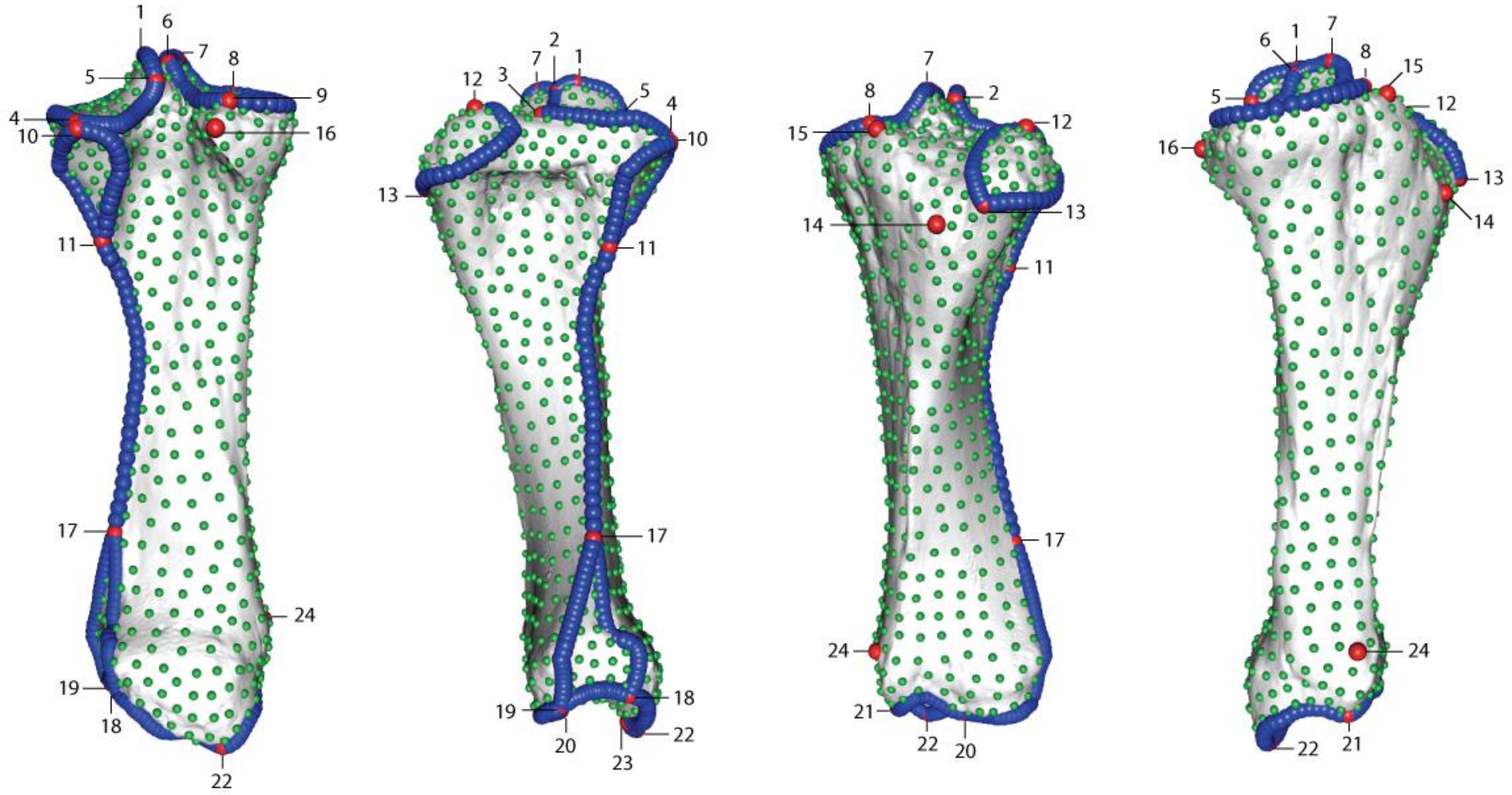

Figure S1K: Location of anatomical landmarks (red spheres), curve sliding (blue spheres) and surface sliding (green spheres) semi-landmarks placed on the tibia. From left to right: caudal, lateral, cranial and medial views. Numbers refer to anatomical landmarks designation detailed in Table S1J.

LM	Designation
**1**	Most proximal point of the proximal tibio-fibular synostosis surface
**2**	Most caudo-medial point of the proximal tibio-fibular synostosis surface
**3**	Most cranio-lateral point of the proximal tibio-fibular synostosis surface
**4**	Most proximal point of the distal tibio-fibular synostosis surface
**5**	Most caudal point of the distal articular facet
**6**	Most distal point of the caudal part of the distal articular facet
**7**	Most distal point of the cranial part of the distal articular facet
**8**	Most cranial point of the distal articular facet
**9**	Distal tip of the caudal ridge of the lateral malleolar sulcus
**10**	Distal tip of the cranial ridge of the lateral malleolar sulcus
**11**	Most lateral point of the cranial ridge of the lateral malleolar sulcus
**12**	Most disto-medial point of the proximal epiphysis = end of the latero-caudal crest

Table S1L: Designation of anatomical landmarks on the fibula.

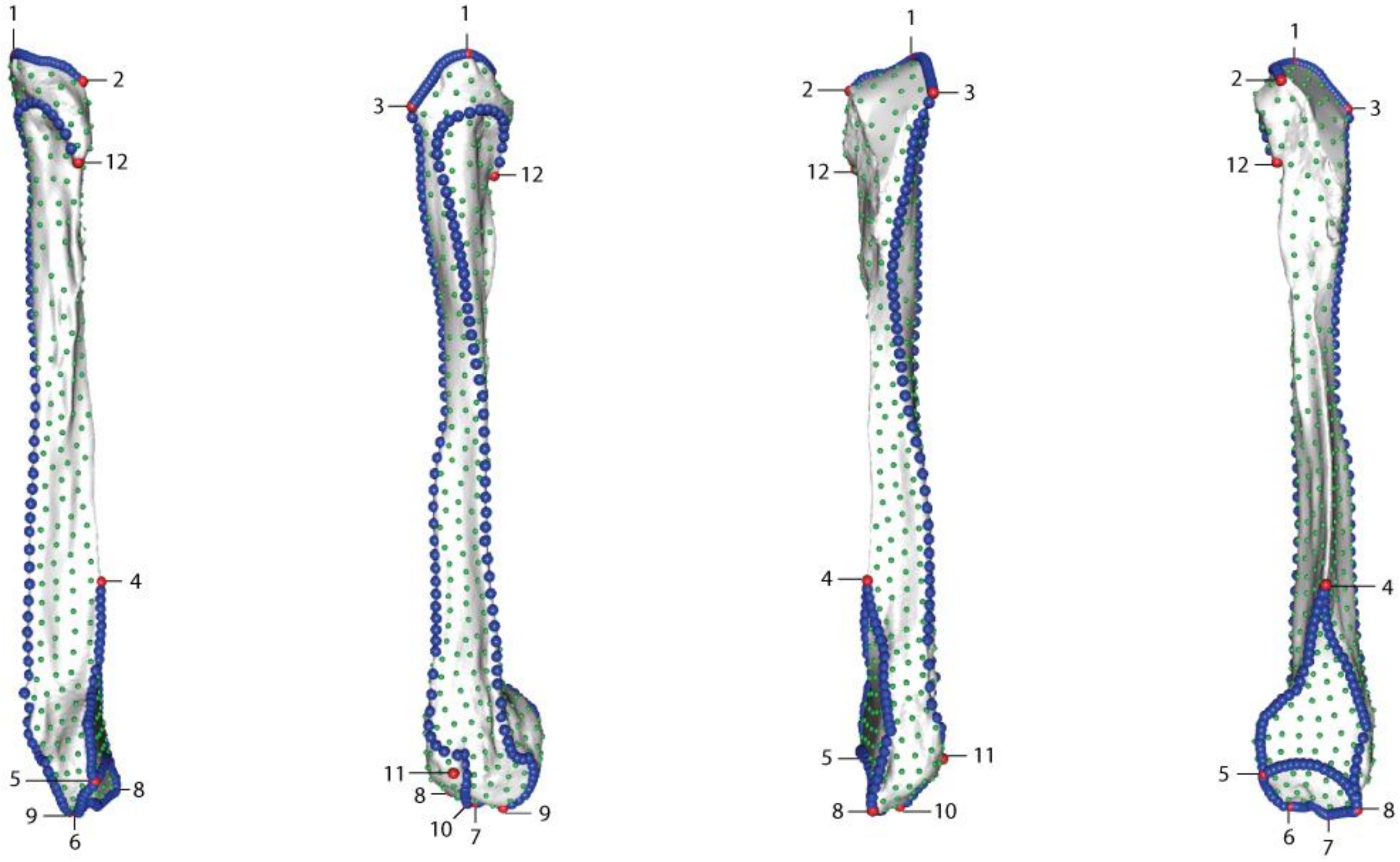

Figure S1M: Location of anatomical landmarks (red spheres), curve sliding (blue spheres) and surface sliding (green spheres) semi-landmarks placed on the fibula. From left to right: caudal, lateral, cranial and medial views. Numbers refer to anatomical landmarks designation detailed in Table S1L.
