# Supplementary material for: Interspecific variation in the limb long bones among modern rhinoceroses—extent and drivers
Source: PeerJ. 2019 Sep 26;7:e7647. doi: 10.7717/peerj.7647 (PMC6766374; doi:10.7717/peerj.7647)
Supplement: Supplemental Information 2 — List of the studied specimens with accession numbers, skeletal composition, sex, age class, condition and 3D acquisition details. [file peerj-07-7647-s002.docx]

The Supplemental Table below provides the details regarding the institution, the number, the available bones, the sex, the age and the condition, as well as the method of 3D acquisition of the data. All the studied specimens are stored in their respective institution.

| **Taxon** | **Institution** | **Specimen number** | **H** | **R** | **U** | **Fe** | **T** | **Fi** | **Sex** | **Age** | **Condition** | **3D acquisition** |
| --- | --- | --- | --- | --- | --- | --- | --- | --- | --- | --- | --- | --- |
| *Ceratotherium simum** | NHMUK | ZD 2018.143 | X | X | X | X | X | X | U | A | U | SS |
| *Ceratotherium simum* | NHMW | 3086 | X | X | X | X | X | X | U | A | W | P |
| *Ceratotherium simum* | RBINS | 19904 | X | X | X | X | X | X | M | S | W | SS |
| *Ceratotherium simum* | RBINS | 35208 | X | X | X | X |  | X | U | A | U | SS |
| *Ceratotherium simum* | RMCA | 1985.32-M-0001 | X | X | X | X | X | X | U | A | W | SS |
| *Ceratotherium simum* | RMCA | RG35146 | X | X | X | X | X | X | M | A | W | SS |
| *Ceratotherium simum* | UCMP | 125000 |  |  |  | X |  |  | U | A | U | CT |
| *Ceratotherium simum* | ZSM | 1912/4199 |  |  |  | X |  |  | U | A | W | SS |
| *Ceratotherium simum* | BICPC | NH.CON.20 | X | X | X | X | X | X | M | S | W | SS |
| *Ceratotherium simum* | BICPC | NH.CON.32 | X | X | X | X | X | X | F | S | W | SS |
| *Ceratotherium simum* | BICPC | NH.CON.37 | X | X |  | X | X | X | F | A | W | SS |
| *Ceratotherium simum* | BICPC | NH.CON.40 | X | X | X | X | X | X | F | S | W | SS |
| *Ceratotherium simum* | BICPC | NH.CON.110 | X | X | X | X | X | X | M | A | W | SS |
| *Ceratotherium simum* | BICPC | NH.CON.112 | X | X | X | X | X | X | M | A | W | SS |
| *Ceratotherium simum* | NMS | NMS.Z.2010.44 | X |  |  | X |  |  | F | A | U | CT |
| *Ceratotherium simum* | MNHN | ZM-MO-2005-297 | X |  |  | X | X | X | M | A | C | SS |
| *Dicerorhinus sumatrensis* | MNHN | ZM-AC-1903-300 | X | X | X | X | X | X | M | A | W | SS |
| *Dicerorhinus sumatrensis* | MNHN | ZM-AC-A7967 | X | X | X |  |  |  | F | A | W | SS |
| *Dicerorhinus sumatrensis* | NHMUK | ZD 1879.6.14.2 | X | X | X | X | X | X | M | A | W | SS |
| *Dicerorhinus sumatrensis* | NHMUK | ZD 1894.9.24.1 | X | X | X | X | X | X | U | A | W | SS |
| *Dicerorhinus sumatrensis* | NHMUK | ZD 1931.5.28.1 | X | X | X | X | X | X | M | S | W | SS |
| *Dicerorhinus sumatrensis* | NHMUK | ZE 1948.12.20.1 | X | X | X | X | X | X | U | A | U | SS |
| *Dicerorhinus sumatrensis* | NHMUK | ZE 1949.1.11.1 | X | X | X | X | X | X | U | A | W | SS |
| *Dicerorhinus sumatrensis* | NHMUK | ZD 2004.23 | X |  |  | X | X | X | U | A | W | SS |
| *Dicerorhinus sumatrensis* | NHMW | 1500 |  |  |  | X | X | X | M | A | U | P |
| *Dicerorhinus sumatrensis* | NHMW | 3082 | X | X | X | X | X | X | U | A | U | P |
| *Dicerorhinus sumatrensis* | NHMW | 29568 |  | X | X | X |  | X | U | S | U | P |
| *Dicerorhinus sumatrensis* | RBINS | 1204 | X | X | X | X | X | X | M | A | W | SS |
| *Dicerorhinus sumatrensis* | UMZC | H.6392 | X |  |  |  |  |  | U | A | U | CT |
| *Dicerorhinus sumatrensis* | ZSM | 1908/571 | X | X |  | X | X | X | M | A | U | SS |
| *Diceros bicornis* | CCEC | 50002040 | X |  |  | X | X | X | U | A | W | SS |
| *Diceros bicornis* | CCEC | 50002044 |  | X |  | X |  |  | U | S | U | SS |
| *Diceros bicornis* | CCEC | 50002045 |  |  |  | X |  |  | U | S | W | SS |
| *Diceros bicornis* | CCEC | 50002046 | X | X | X |  | X | X | U | S | U | SS |
| *Diceros bicornis* | CCEC | 50002047 |  | X | X |  | X | X | U | A | U | SS |
| *Diceros bicornis* | MNHN | ZM-AC-1936-644 | X | X | X | X | X | X | F | S | U | SS |
| *Diceros bicornis* | MNHN | ZM-AC-1944-278 | X |  |  | X | X | X | M | A | C | SS |
| *Diceros bicornis* | MNHN | ZM-AC-1974-124 |  |  |  | X | X | X | F | A | C | SS |
| *Diceros bicornis* | RBINS | 9714 | X | X | X | X | X | X | F | A | W | SS |
| *Diceros bicornis* | RMCA | RG2133 | X | X | X | X | X | X | M | S | W | SS |
| *Diceros bicornis* | UCMP | 9856 |  |  |  |  | X |  | U | A | U | CT |
| *Diceros bicornis* | ZSM | 1961/186 | X | X | X | X | X | X | M | S | U | SS |
| *Diceros bicornis* | ZSM | 1961/187 | X | X | X | X | X | X | M | S | U | SS |
| *Diceros bicornis* | ZSM | 1962/166 | X | X | X | X | X |  | F | S | U | SS |
| *Rhinoceros sondaicus* | CCEC | 50002041 | X | X | X | X | X | X | U | A | W | SS |
| *Rhinoceros sondaicus* | CCEC | 50002043 | X | X | X | X |  |  | U | A | W | SS |
| *Rhinoceros sondaicus* | MNHN | ZM-AC-A7970 | X | X | X | X | X | X | U | A | U | SS |
| *Rhinoceros sondaicus* | MNHN | ZM-AC-A7971 | X | X | X | X | X | X | U | A | W | SS |
| *Rhinoceros sondaicus* | NHMUK | ZD 1861.3.11.1 | X | X | X | X | X | X | U | S | W | SS |
| *Rhinoceros sondaicus* | NHMUK | ZD 1871.12.29.7 | X | X | X | X | X | X | M | A | W | SS |
| *Rhinoceros sondaicus* | NHMUK | ZD 1921.5.15.1 | X | X | X | X | X | X | F | S | W | SS |
| *Rhinoceros sondaicus* | RBINS | 1205F | X | X | X | X | X | X | U | S | W | SS |
| *Rhinoceros unicornis*** | MNHN | ZM-AC-1885-734 | X | X | X | X | X |  | U | A | W | SS |
| *Rhinoceros unicornis* | MNHN | ZM-AC-1932-49 | X |  |  |  | X | X | U | S | U | SS |
| *Rhinoceros unicornis* | MNHN | ZM-AC-1960-59 | X | X | X | X | X | X | M | A | C | SS |
| *Rhinoceros unicornis* | MNHN | ZM-AC-1967-101 | X | X | X | X | X |  | F | A | C | SS |
| *Rhinoceros unicornis* | NHMUK | ZD 1884.1.22.1.2 | X | X | X | X | X | X | F | A | W | SS |
| *Rhinoceros unicornis* | NHMUK | ZE 1950.10.18.5 | X | X | X | X | X | X | M | A | W | SS |
| *Rhinoceros unicornis* | NHMUK | ZE 1961.5.10.1 | X | X | X | X | X | X | M | A | W | SS |
| *Rhinoceros unicornis** | NHMUK | ZD 1972.822 | X | X | X | X | X | X | U | A | U | SS |
| *Rhinoceros unicornis* | RBINS | 1208 | X | X | X | X | X | X | F | A | C | SS |
| *Rhinoceros unicornis* | RBINS | 33382 | X | X | X | X | X | X | U | A | U | SS |

**Supplemental Table: List of the studied specimens with skeletal composition, sex, age class, condition and 3D acquisition details.**

**Abbreviations: Bones – H**: humerus; **R**: radius; **U**: ulna; **Fe**: femur; **T**: tibia; **Fi**: fibula. **Sex:** **F**: female; **M**: male; **U**: unknown. **Age – A**: adult; **Sa**: sub-adult. **Condition – W**: wild; **C**: captive; **U**: unknown. **3D acquisition – SS**: surface scanner; **P**: photogrammetry; **CT**: CT-scan. **Institutional codes: BICPC**: Powell Cotton Museum, Birchington-on-Sea. **CCEC**: Centre de Conservation et d’Étude des Collections, Musée des Confluences, Lyon. **MHNT:** Muséum d’Histoire Naturelle de Toulouse, Toulouse. **MNHN:** Muséum National d’Histoire Naturelle, Paris. **NHMUK:** Natural History Museum, London. **NHMW:** Naturhistorisches Museum Wien, Vienna. **NMS:** National Museums Scotland, Edinburgh. **RBINS:** Royal Belgian Institute of Natural Sciences, Brussels. **RMCA:** Royal Museum for Central Africa, Tervuren. **UCMP:** University of California Museum of Paleontology, Berkeley. **UMZC:** University Museum of Zoology Cambridge, Cambridge. **ZSM:** Zoologische Staatssammlung München, Munich. * Specimens NHMUK ZD 2018.143 and NHMUK ZD 1972.822 were determined by ourselves during the visit of the collections on the basis of morphological observations and measurements on the post-cranial elements. These determinations were later confirmed by our shape analysis. ** The specimen MNHN-ZM-AC-1885-734 was previously determined as *Rhinoceros sondaicus* based on a supposed Javan origin. The observations made on both long bones and tarsal elements led us to consider this individual as an Indian rhino (*Rhinoceros unicornis*). This attribution was later confirmed by our shape analysis.
