# Supplementary figures and images for: Interspecific variation in the limb long bones among modern rhinoceroses—extent and drivers
Source: PeerJ. 2019 Sep 26;7:e7647. doi: 10.7717/peerj.7647 (PMC6766374; doi:10.7717/peerj.7647)

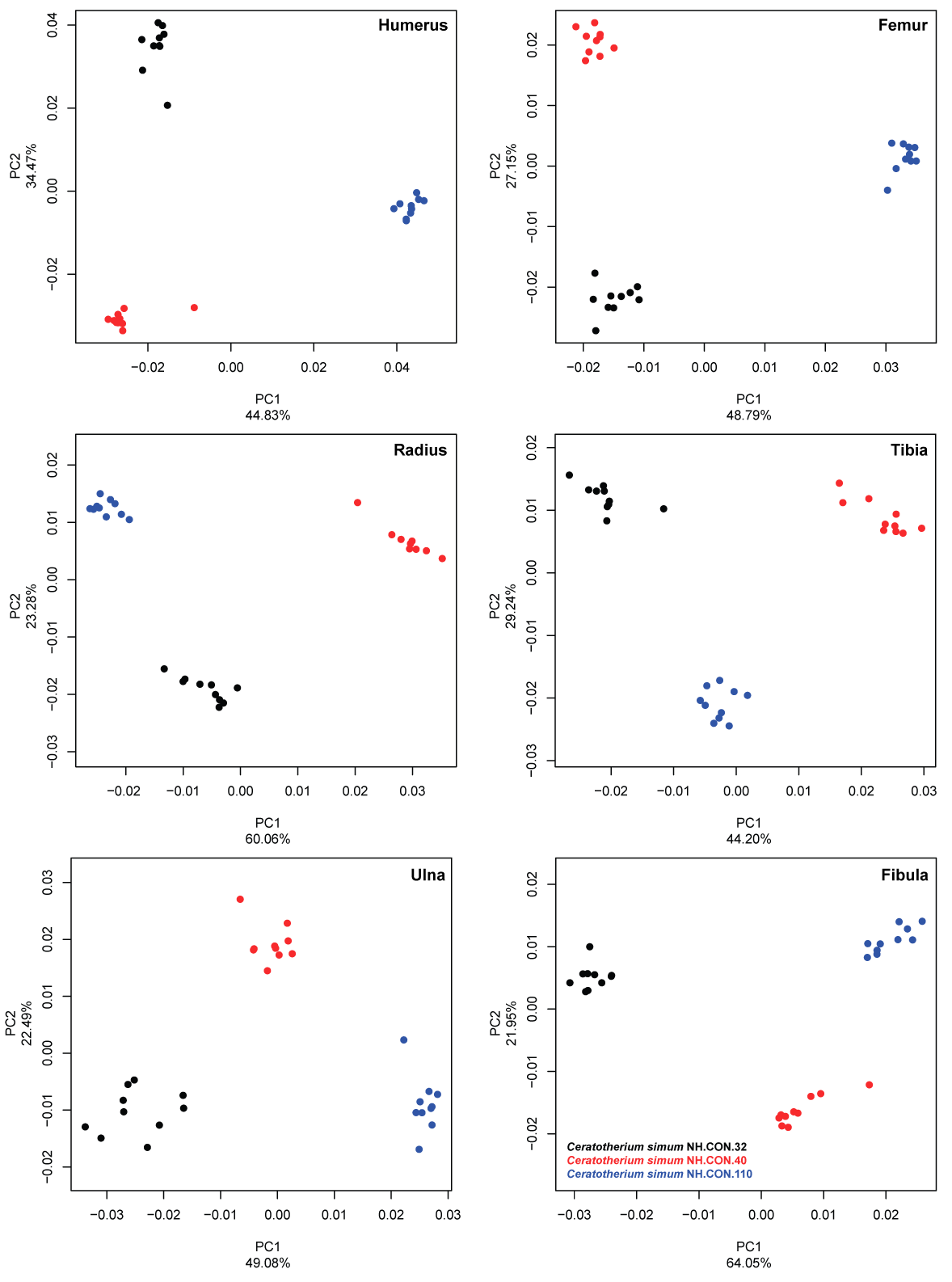

Supplement: Supplemental Information 3 — Each landmark configuration was digitized ten times on three specimens of Ceratotherium simum chosen to display the fewer morphological difference as possible. Each color corresponds to a specimen. For each bone, the inter-specimen variation is lower than the intra-specimen error due to differences between landmark digitization. We concluded to the relevance of our anatomical landmark configuration to describe shape variation within our sample. [file peerj-07-7647-s003.png]

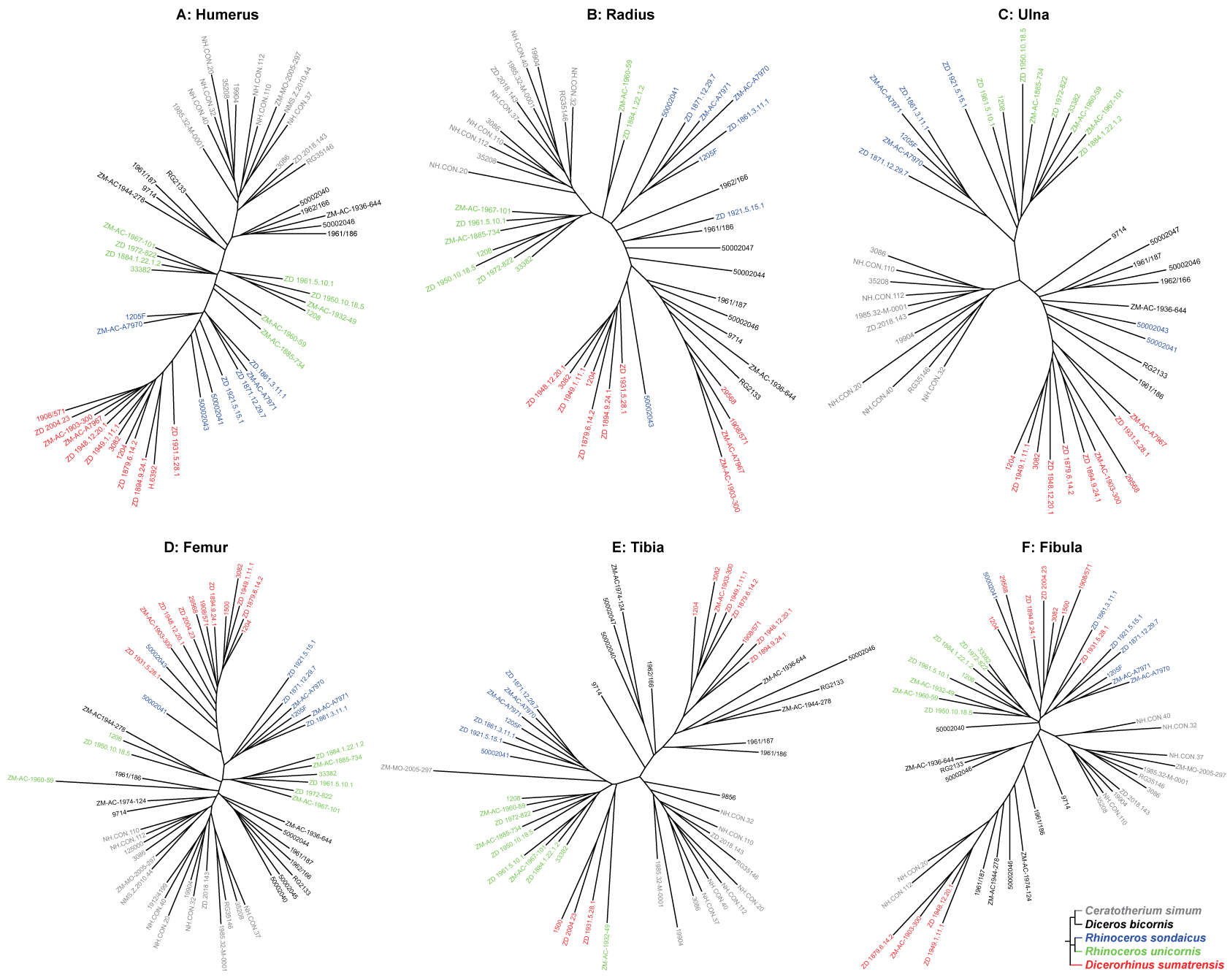

Supplement: Supplemental Information 5 — A: Humerus; B: Radius; C: Ulna; D: Femur; E: Tibia; F: Fibula. Specimen codes are given following the Table 2. [file peerj-07-7647-s005.png]

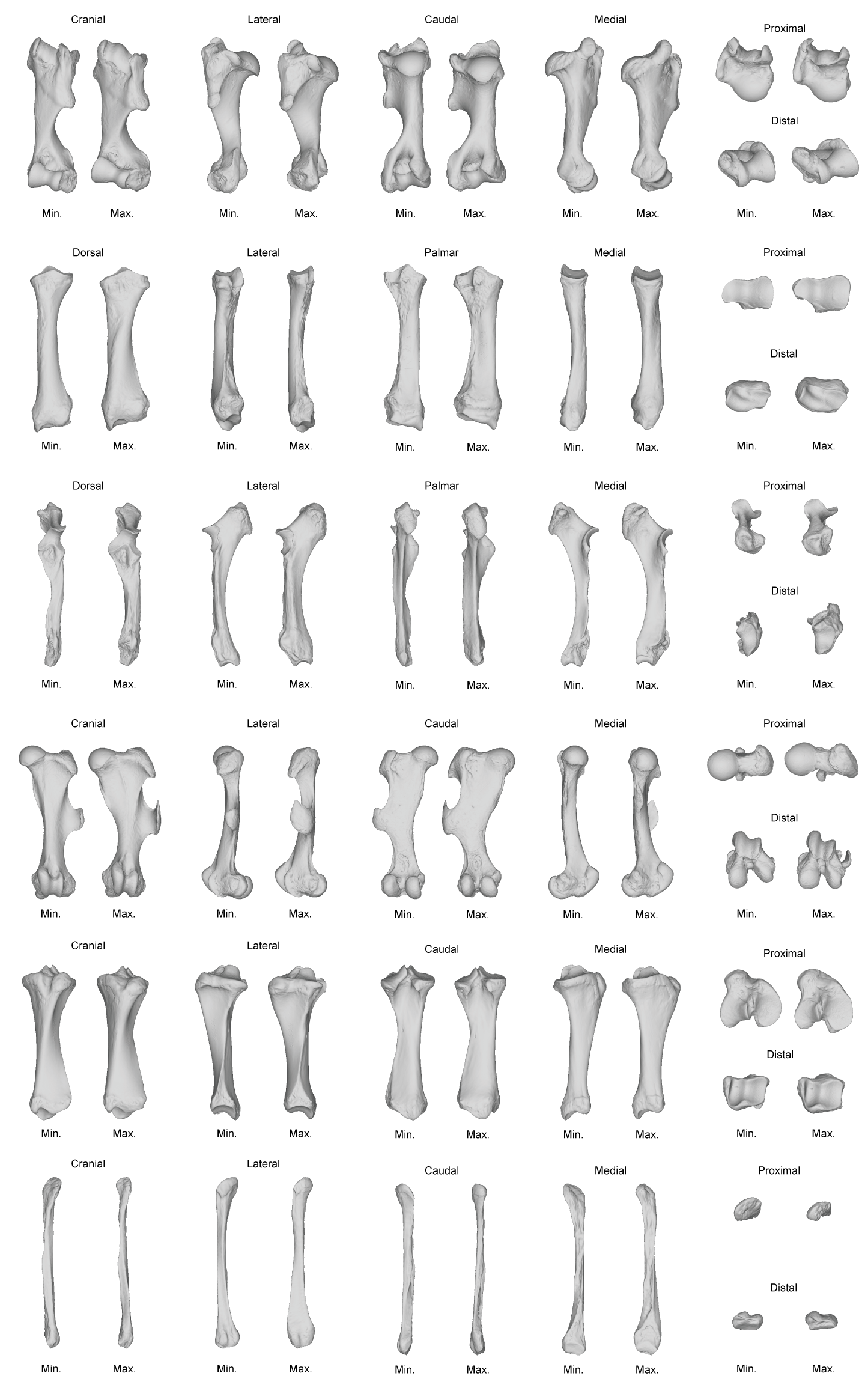

Supplement: Supplemental Information 7 [file peerj-07-7647-s007.png]

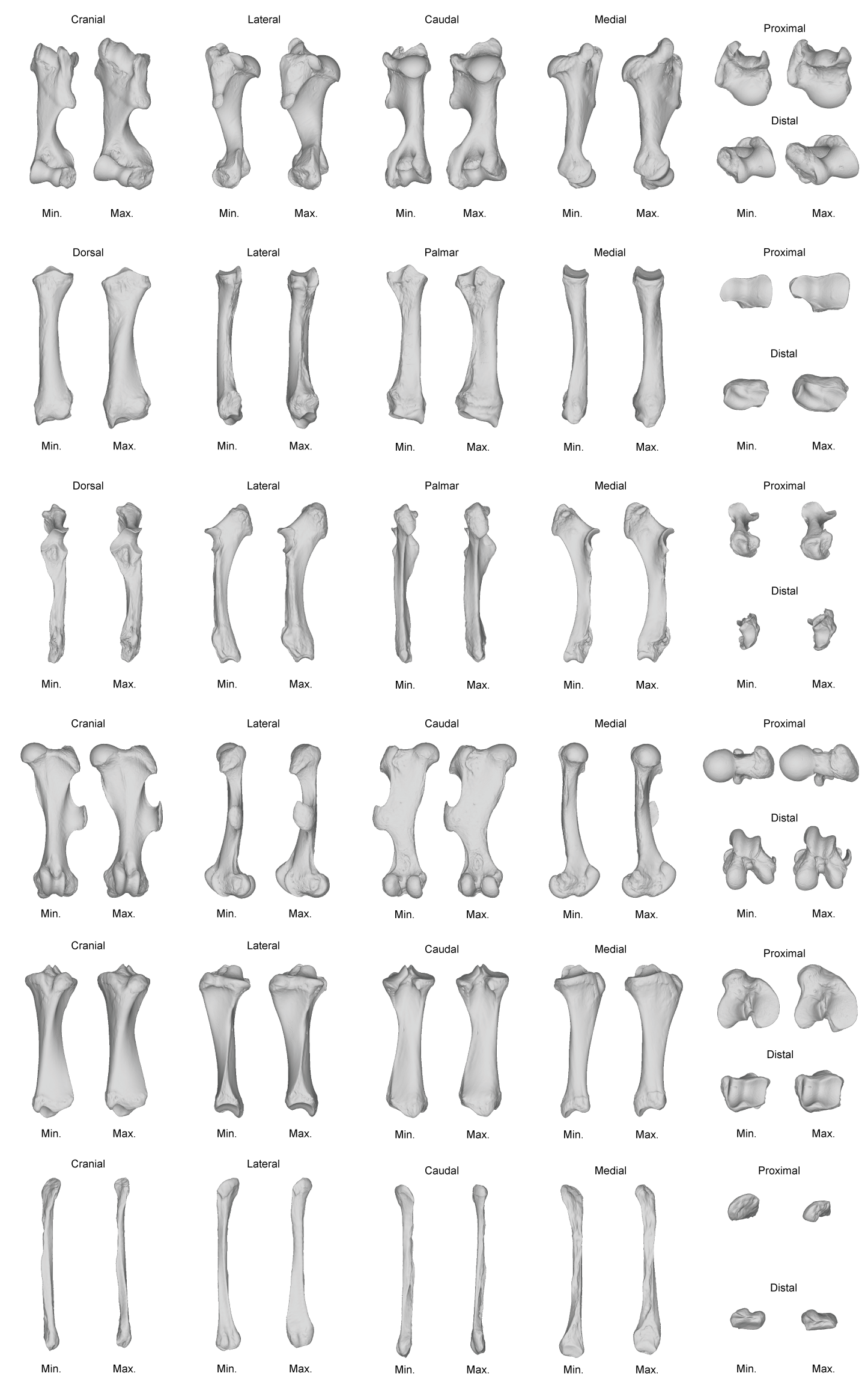

Supplement: Supplemental Information 8 [file peerj-07-7647-s008.png]
