# Supplementary material for: Interspecific variation in the limb long bones among modern rhinoceroses—extent and drivers
Source: PeerJ. 2019 Sep 26;7:e7647. doi: 10.7717/peerj.7647 (PMC6766374; doi:10.7717/peerj.7647)
Supplement: Supplemental Information 4 [file peerj-07-7647-s004.pdf]

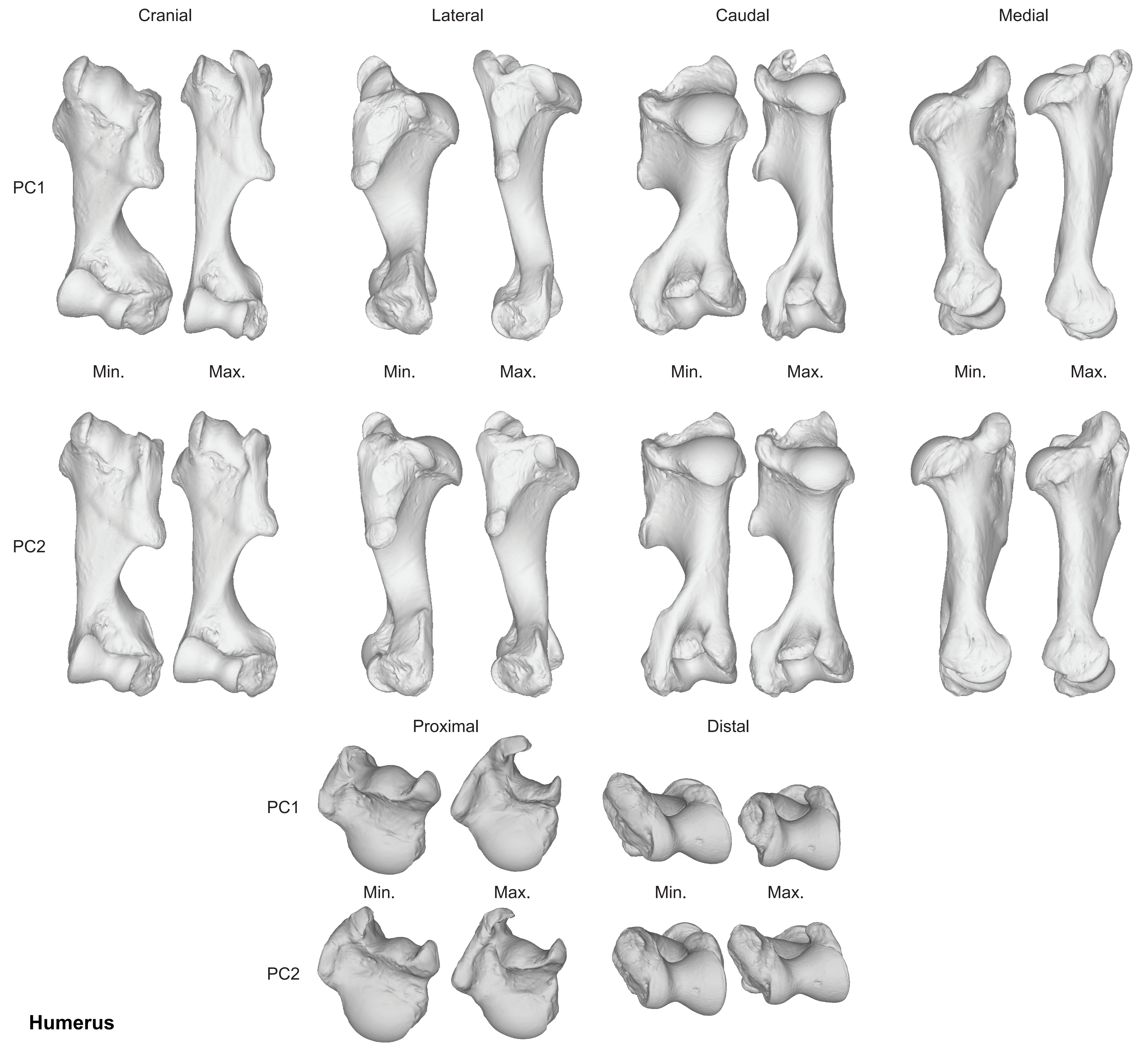
Cranial
Lateral
Caudal
Medial
PC1
Min.
Max.
Min.
Max.
Min.
Max.
Min.
Max.
PC2
Proximal
Distal
PC1
Min.
Max.
Min.
Max.
PC2
Humerus

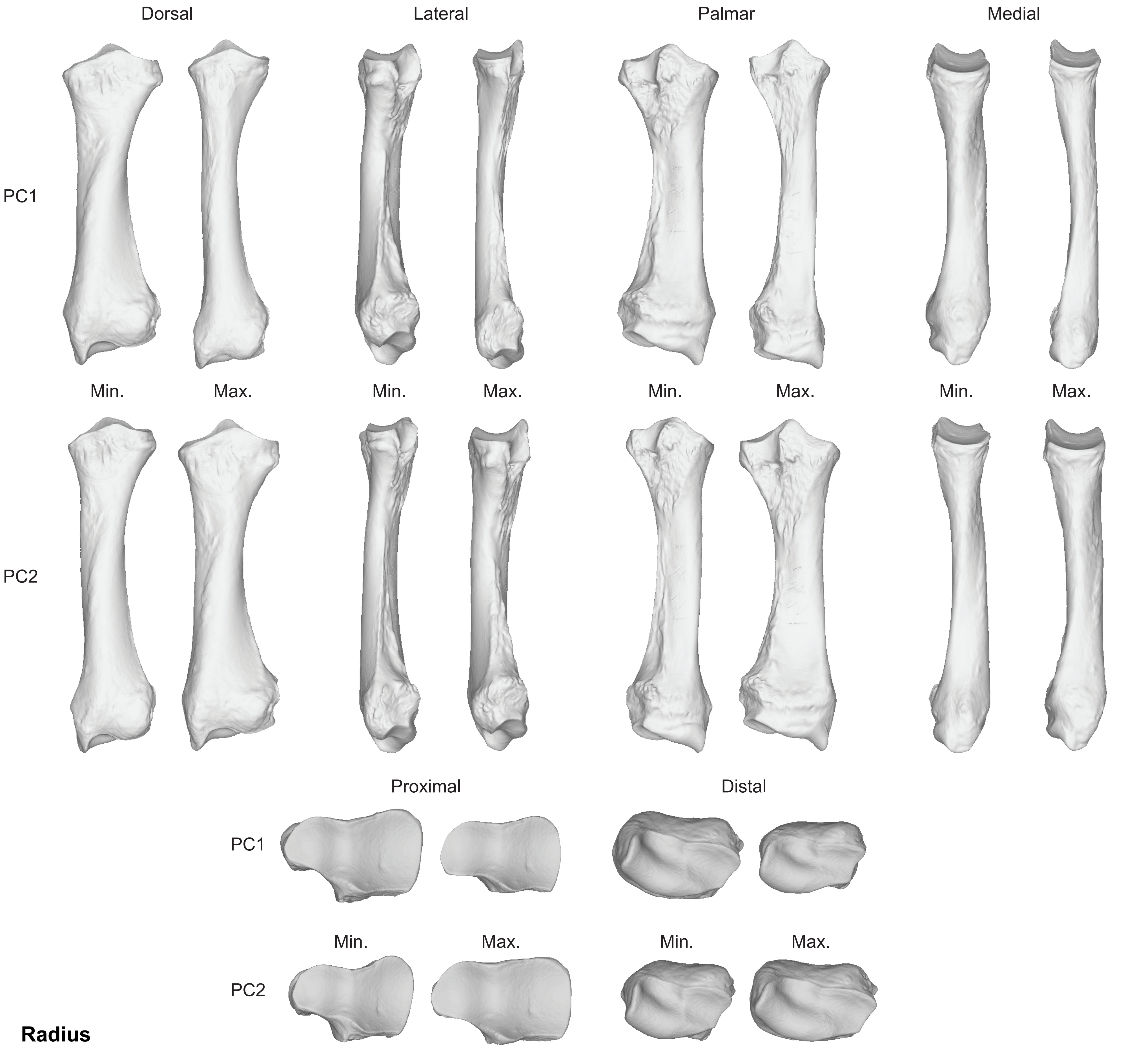

Dorsal
Lateral
Palmar
Medial
PC1
Min.
Max.
Min.
Max.
Min.
Max.
Min.
Max.
PC2
Proximal
Distal
PC1
Min.
Max.
Min.
Max.
PC2
Radius

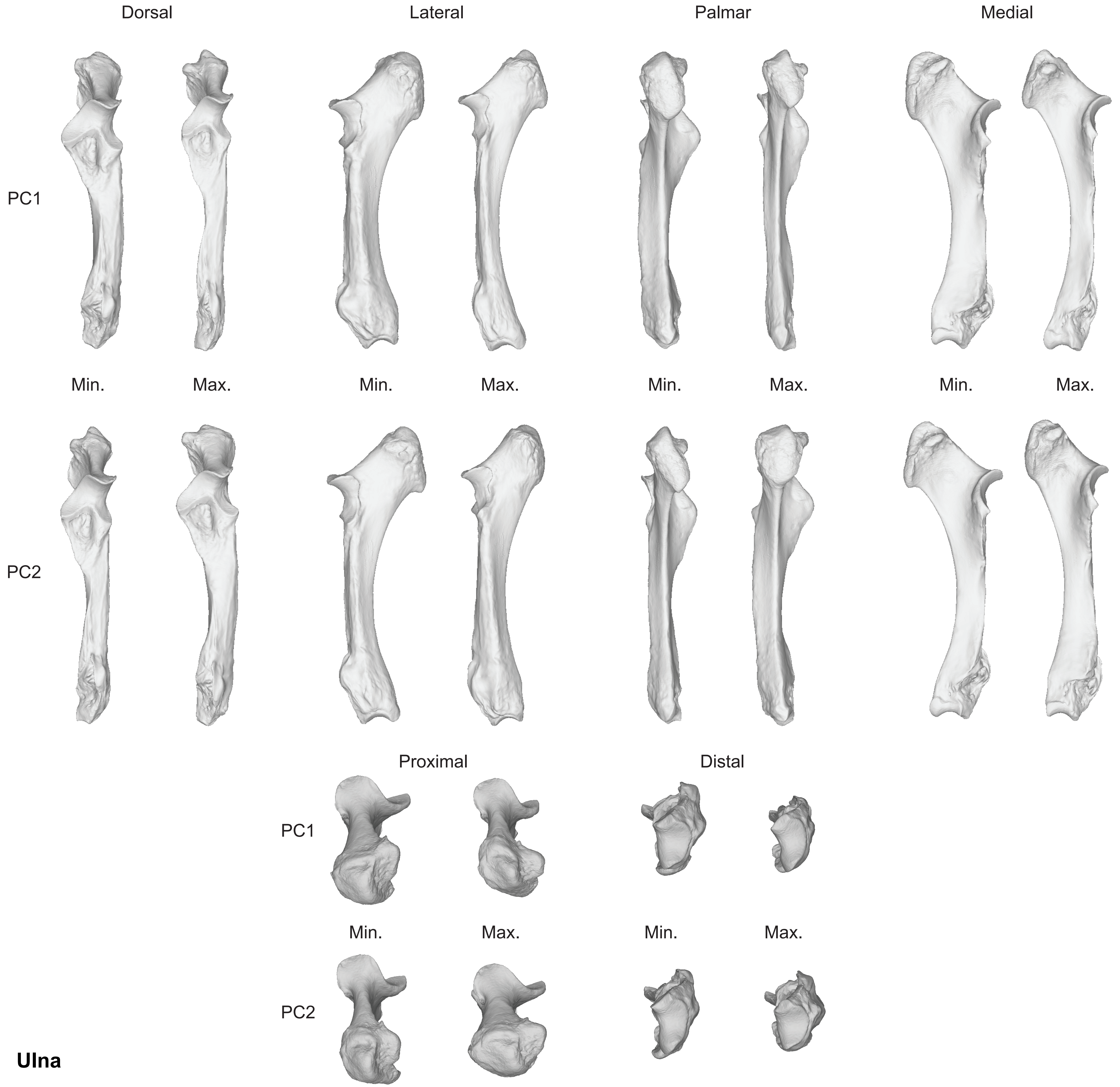

Dorsal
Lateral
Palmar
Medial
PC1
Min.
Max.
Min.
Max.
Min.
Max.
Min.
Max.
PC2
Proximal
Distal
PC1
Min.
Max.
Min.
Max.
PC2
Ulna

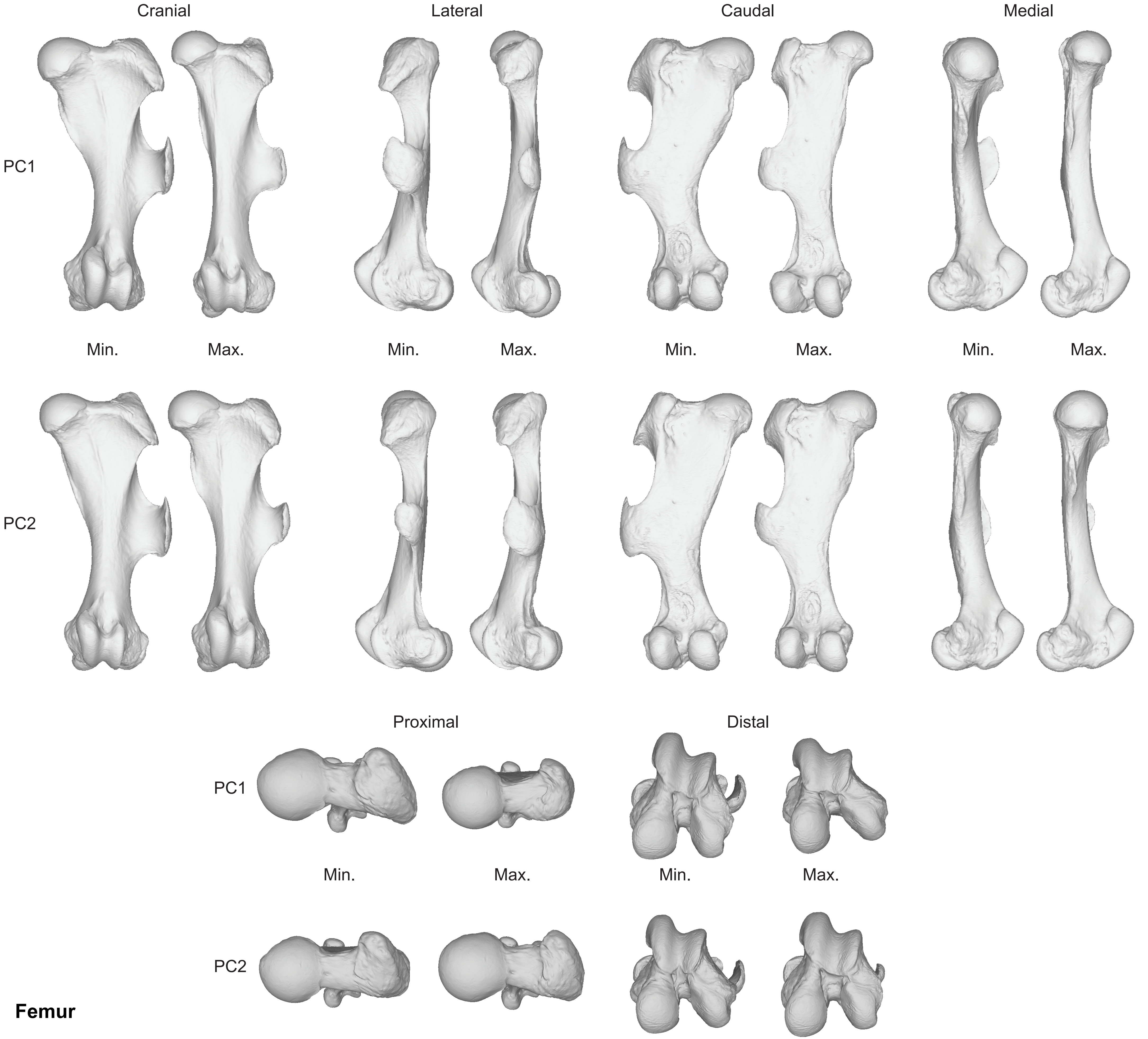

Cranial
Lateral
Caudal
Medial
PC1
Min.
Max.
Min.
Max.
Min.
Max.
Min.
Max.
PC2
Proximal
Distal
PC1
Min.
Max.
Min.
Max.
PC2
Femur

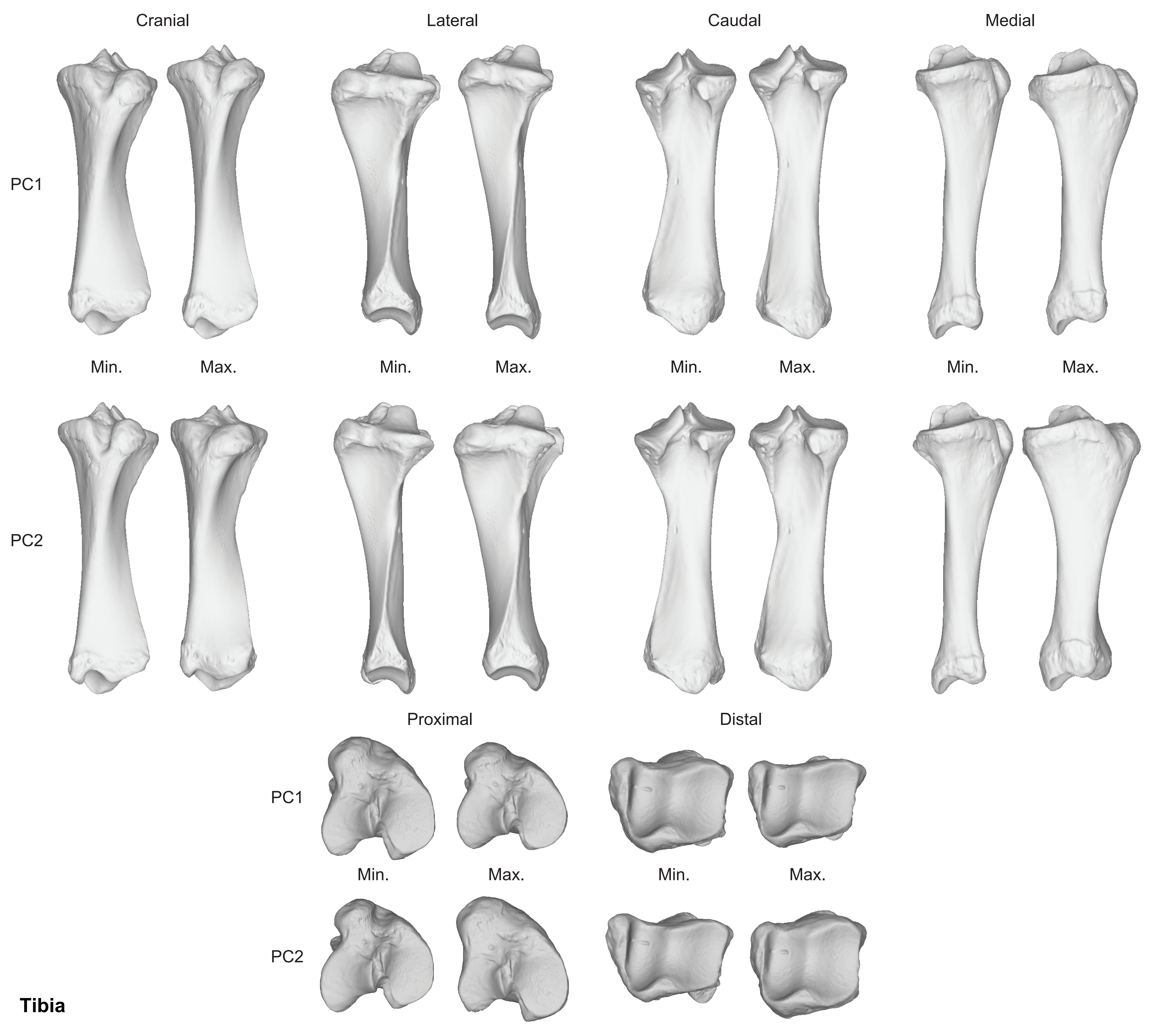

Cranial
Lateral
Caudal
Medial
PC1
Min.
Max.
Min.
Max.
Min.
Max.
Min.
Max.
PC2
Proximal
Distal
PC1
Min.
Max.
Min.
Max.
PC2
Tibia

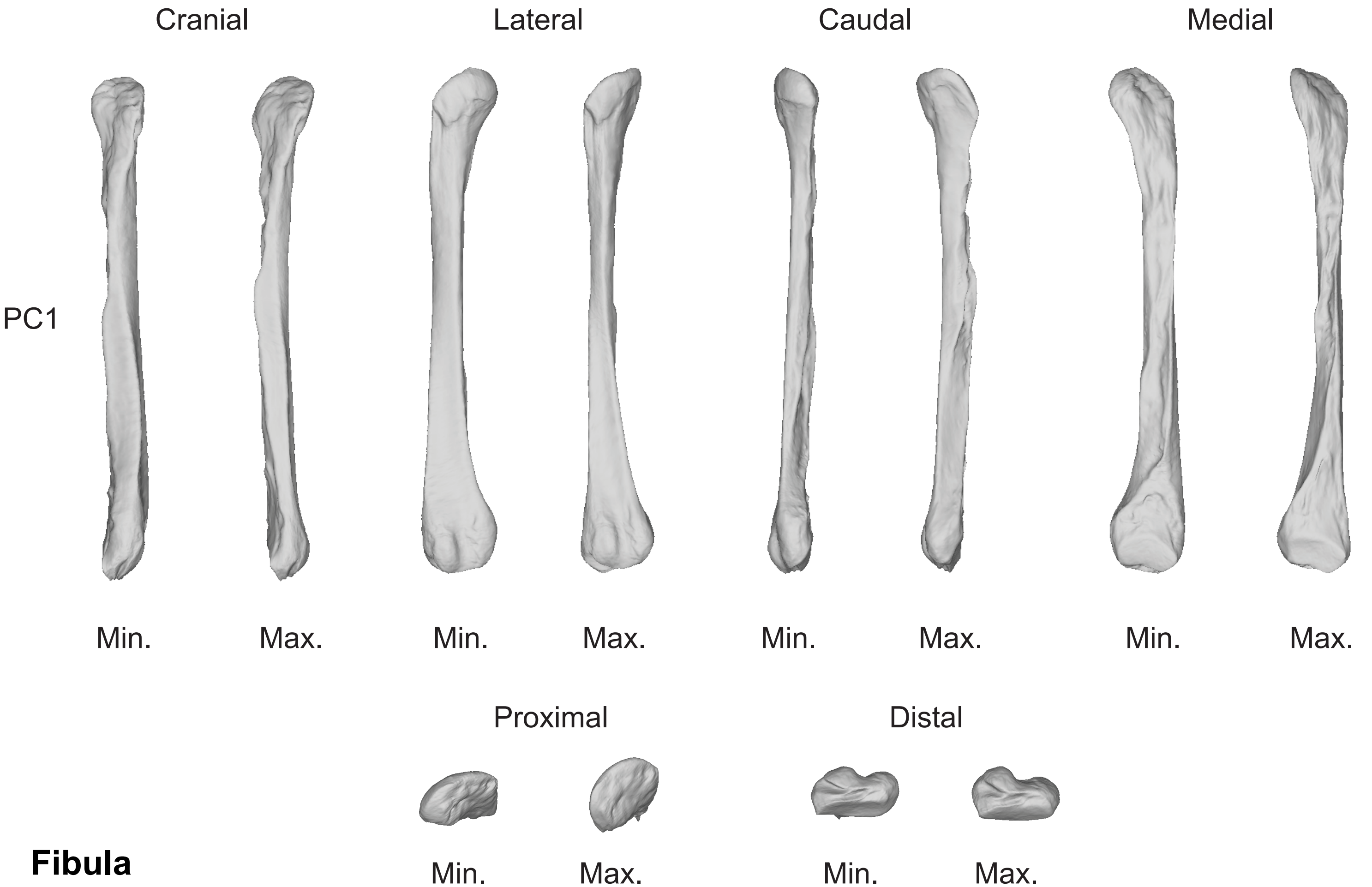
Cranial
Lateral
Caudal
Medial
PC1
Min.
Max.
Min.
Max.
Min.
Max.
Min.
Max.
Proximal
Distal
Min.
Max.
Min.
Max.
Fibula

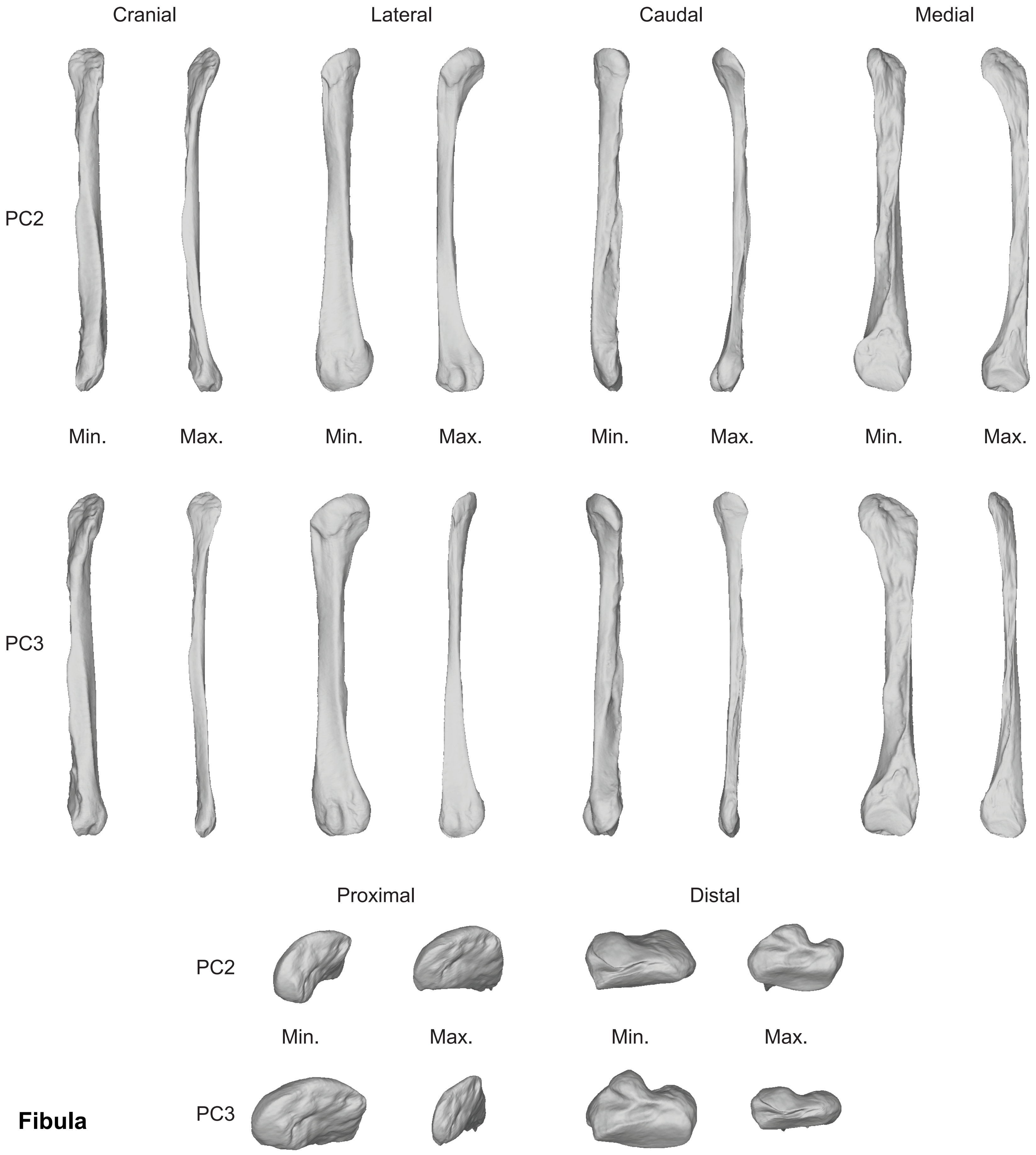

Cranial
Lateral
Caudal
Medial
PC2
Min.
Max.
Min.
Max.
Min.
Max.
Min.
Max.
PC3
Proximal
Distal
PC2
Min.
Max.
Min.
Max.
PC3
Fibula
