# Supplementary material for: Interspecific variation in the limb long bones among modern rhinoceroses—extent and drivers
Source: PeerJ. 2019 Sep 26;7:e7647. doi: 10.7717/peerj.7647 (PMC6766374; doi:10.7717/peerj.7647)
Supplement: Supplemental Information 6 [file peerj-07-7647-s006.pdf]

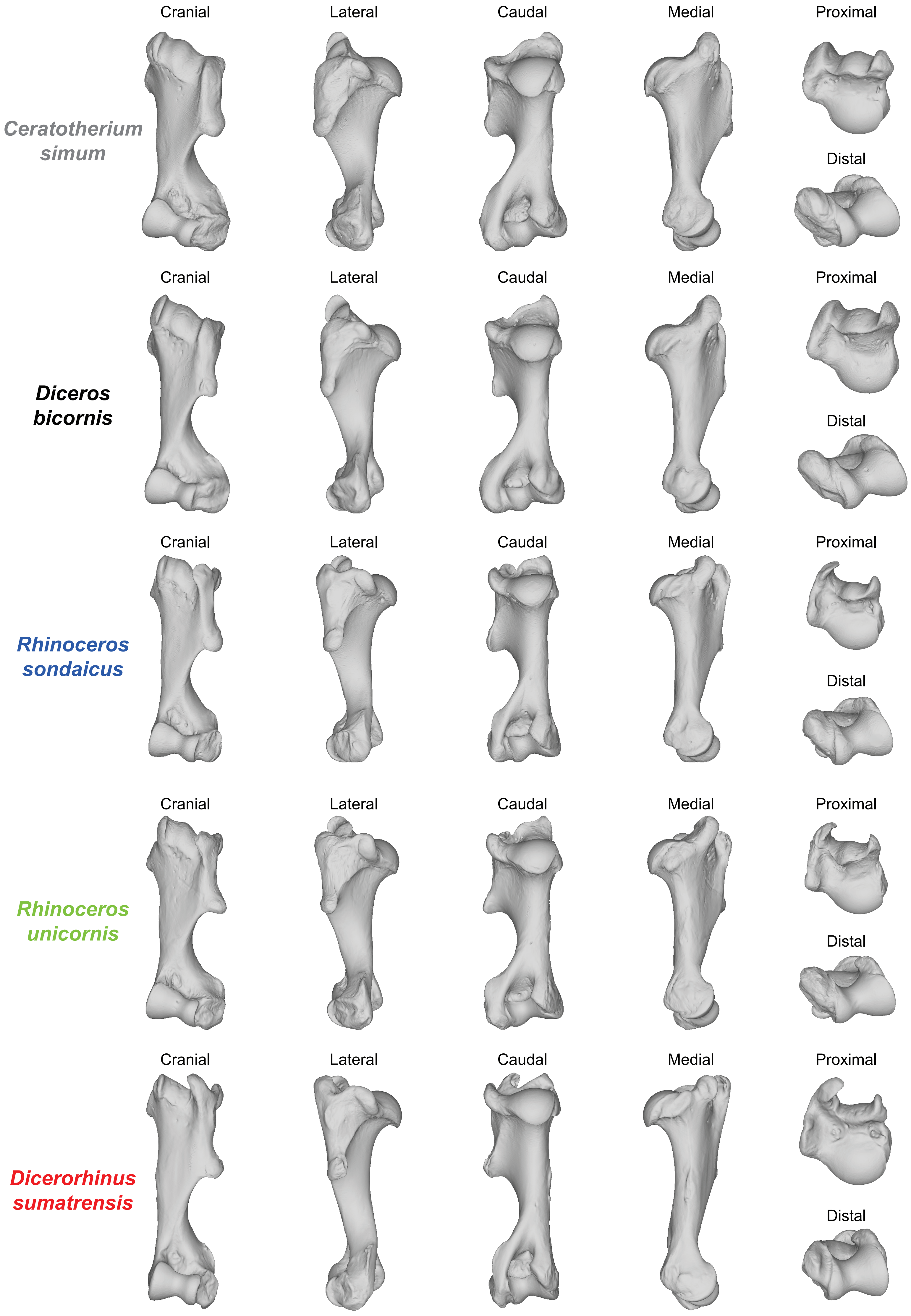

Cranial
Lateral
Caudal
Medial
Proximal
Distal
Ceratotherium simum
Diceros bicornis
Rhinoceros sondaicus
Rhinoceros unicornis
Dicerorhinus sumatrensis

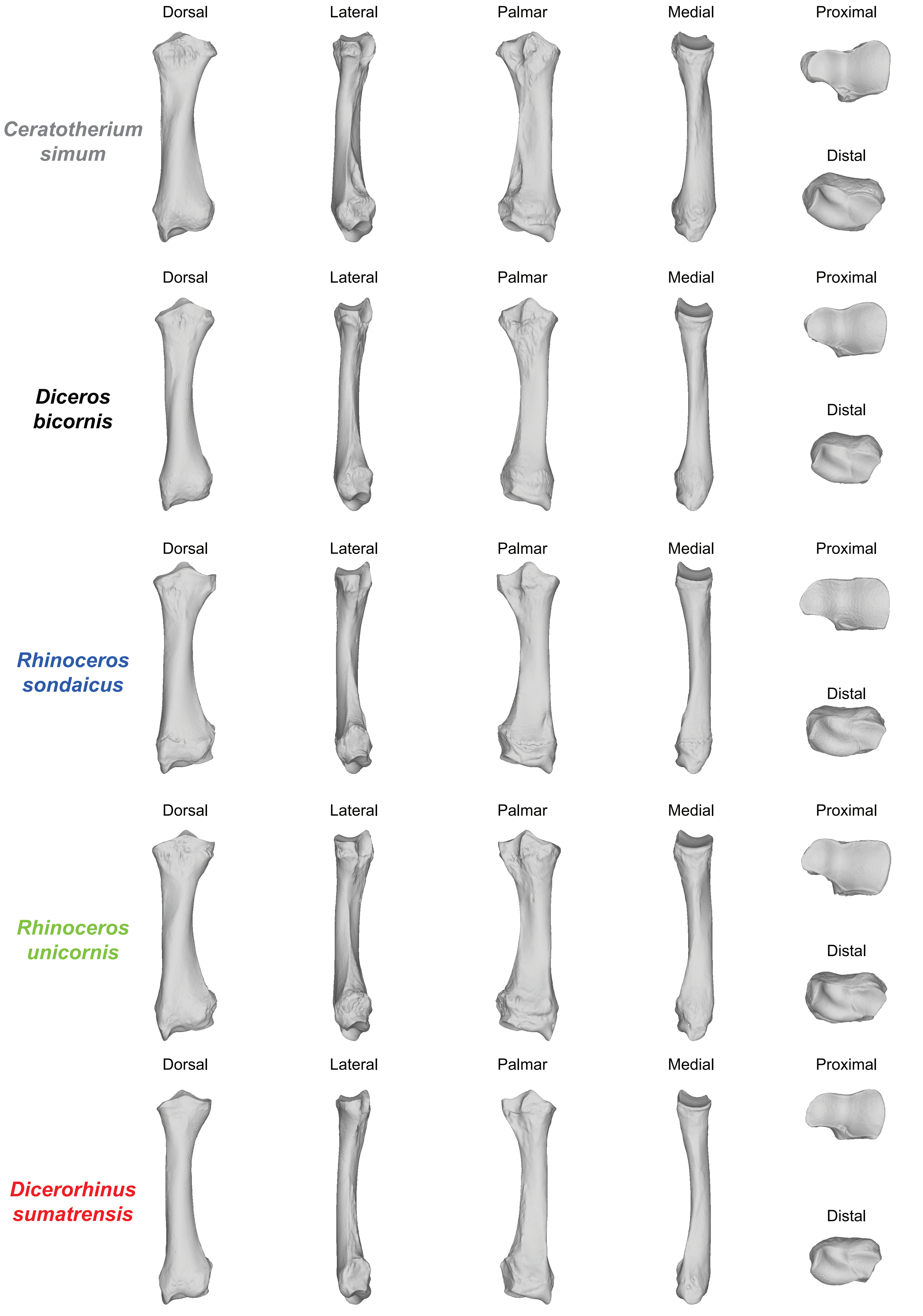
Dorsal
Lateral
Palmar
Medial
Proximal
Distal
Ceratotherium simum
Diceros bicornis
Rhinoceros sondaicus
Rhinoceros unicornis
Dicerorhinus sumatrensis

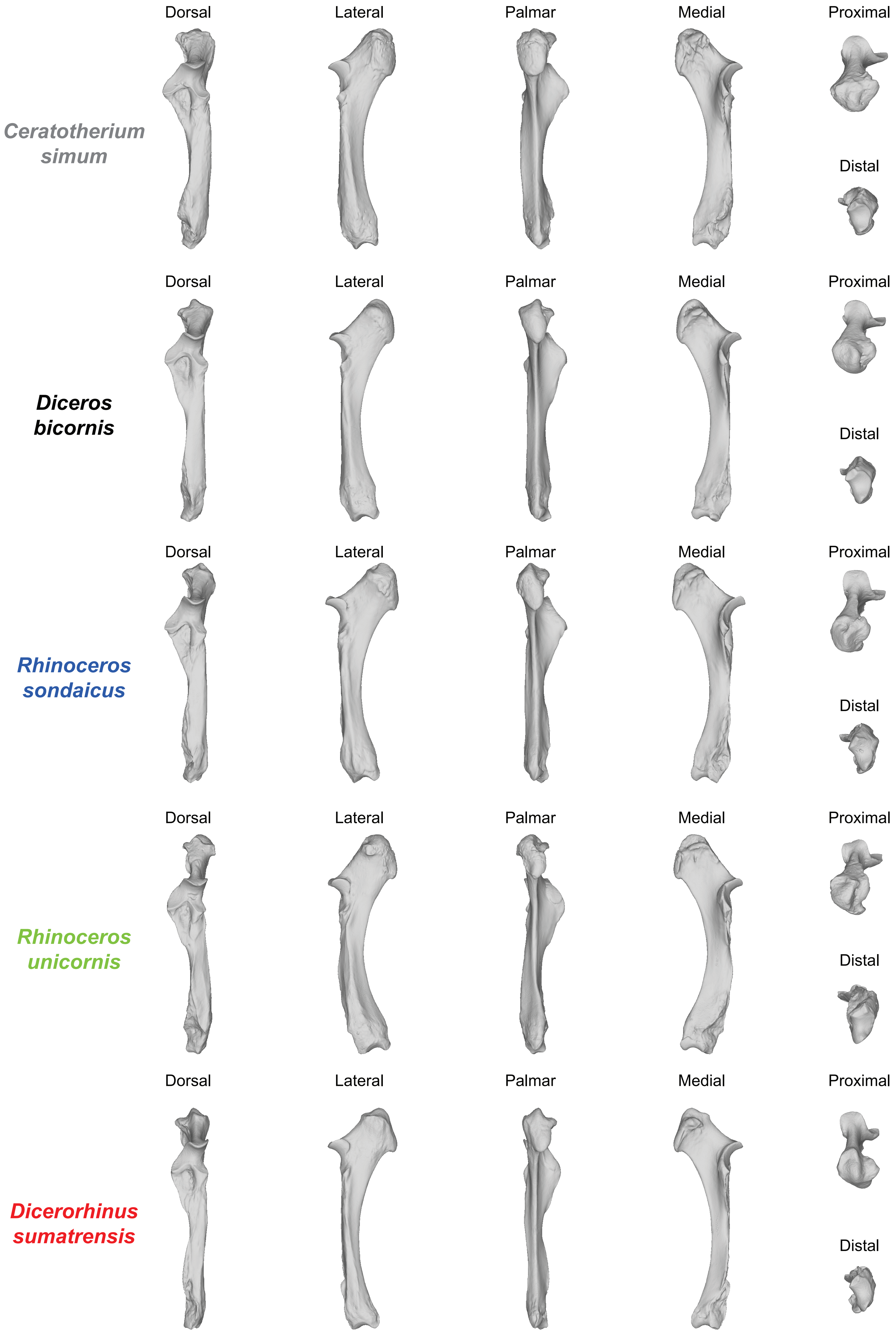
Dorsal
Lateral
Palmar
Medial
Proximal
Distal
Ceratotherium simum
Diceros bicornis
Rhinoceros sondaicus
Rhinoceros unicornis
Dicerorhinus sumatrensis

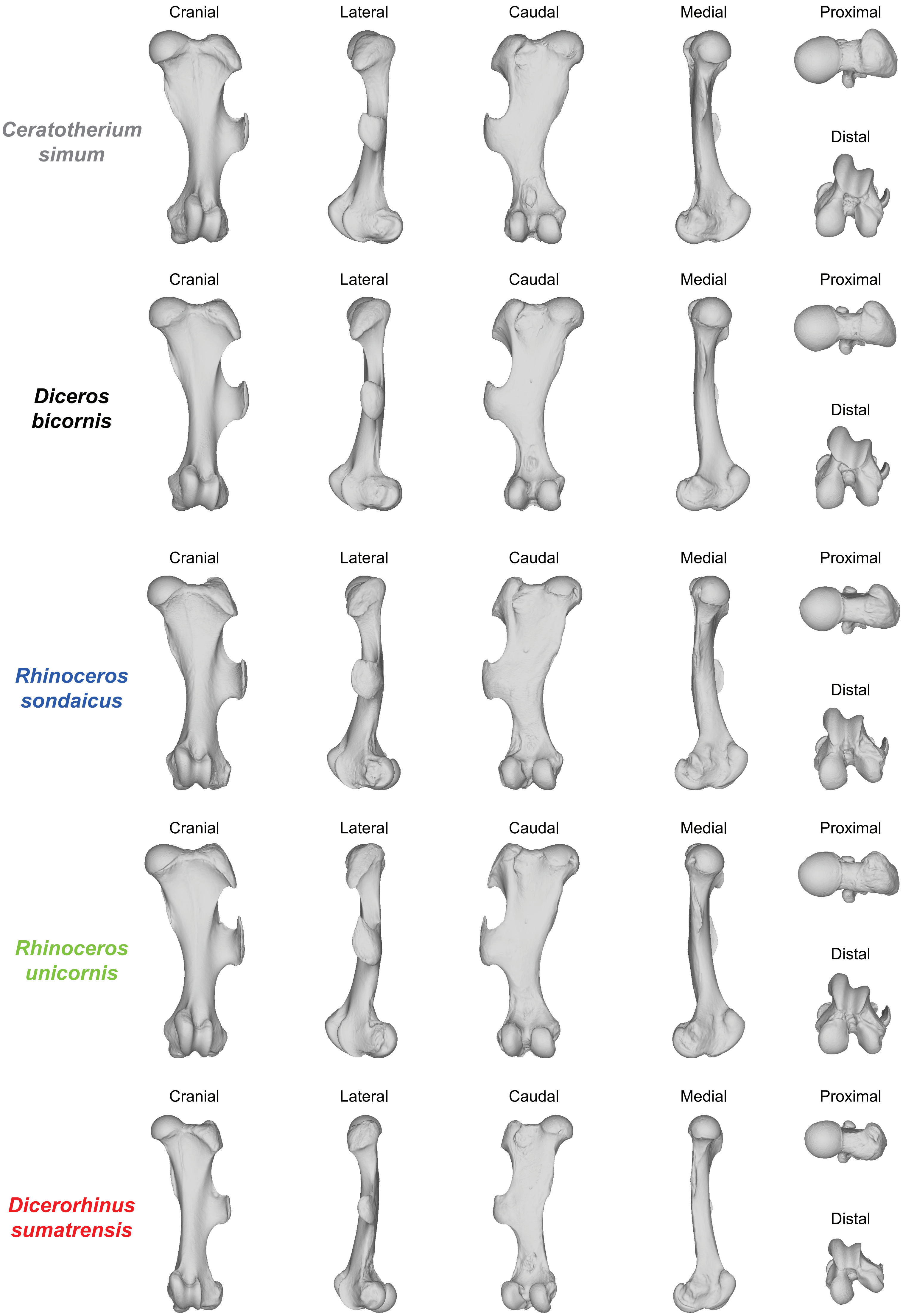
Cranial
Lateral
Caudal
Medial
Proximal
Ceratotherium simum
Distal
Cranial
Lateral
Caudal
Medial
Proximal
Diceros bicornis
Distal
Cranial
Lateral
Caudal
Medial
Proximal
Rhinoceros sondaicus
Distal
Cranial
Lateral
Caudal
Medial
Proximal
Rhinoceros unicornis
Distal
Cranial
Lateral
Caudal
Medial
Proximal
Dicerorhinus sumatrensis
Distal

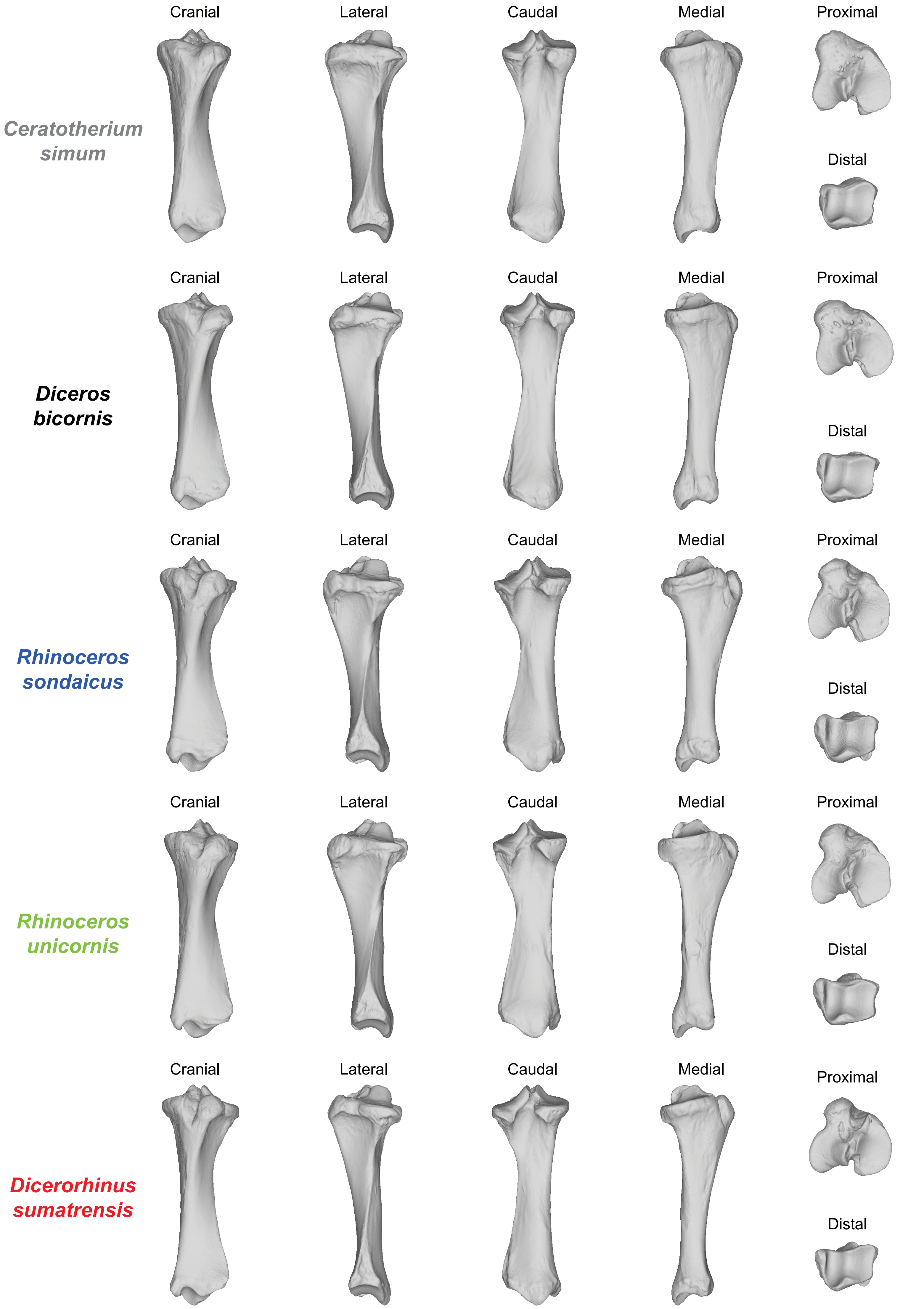

Cranial
Lateral
Caudal
Medial
Proximal
Distal
Ceratotherium simum
Diceros bicornis
Rhinoceros sondaicus
Rhinoceros unicornis
Dicerorhinus sumatrensis

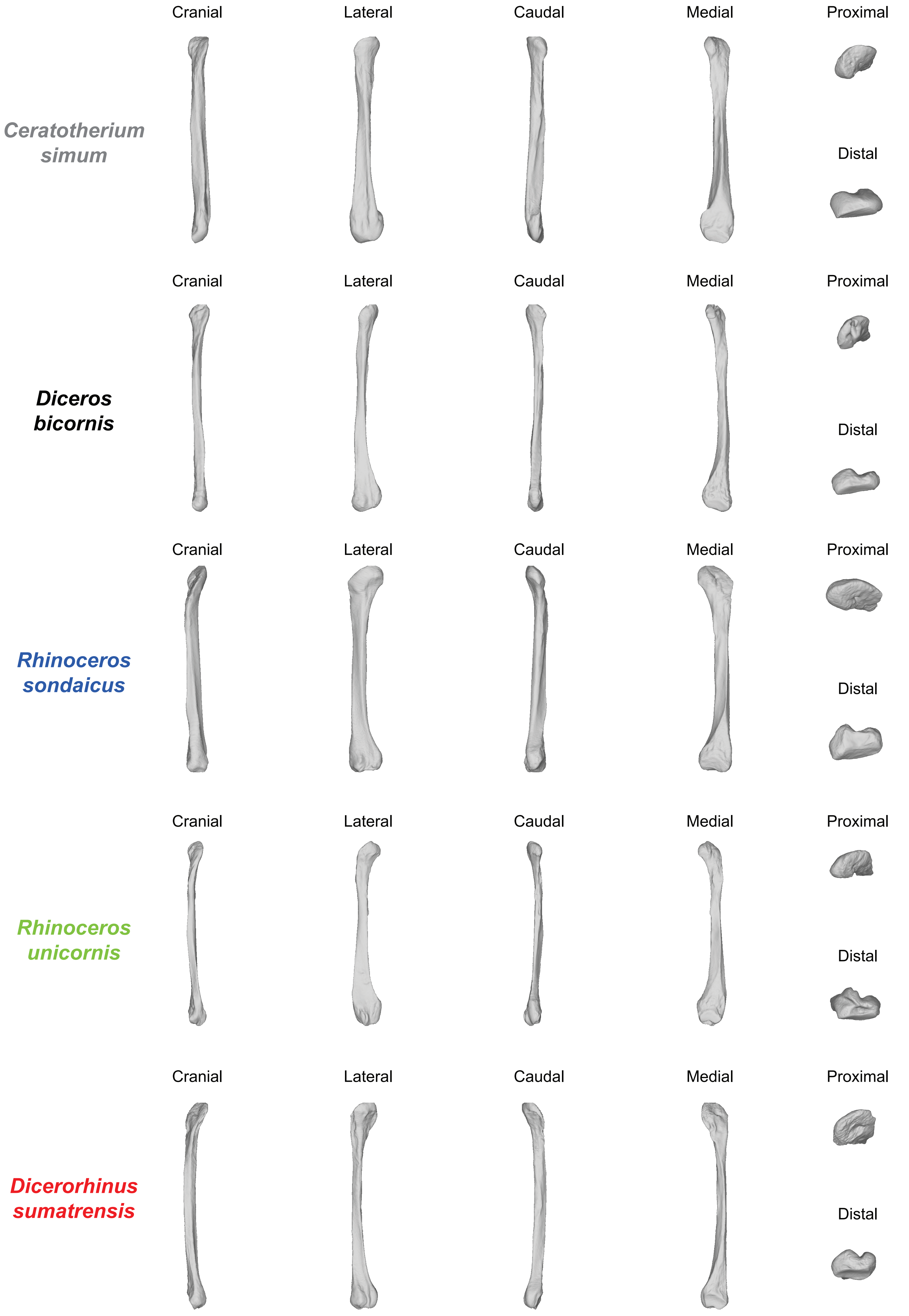
Cranial
Lateral
Caudal
Medial
Proximal
Ceratotherium simum
Distal
Cranial
Lateral
Caudal
Medial
Proximal
Diceros bicornis
Distal
Cranial
Lateral
Caudal
Medial
Proximal
Rhinoceros sondaicus
Distal
Cranial
Lateral
Caudal
Medial
Proximal
Rhinoceros unicornis
Distal
Cranial
Lateral
Caudal
Medial
Proximal
Dicerorhinus sumatrensis
Distal
